# Mutual Activation of Two Radical Trapping Agents: Unusual “Win–Win Synergy” of Resveratrol and TEMPO during Scavenging of dpph^•^ Radical in Methanol

**DOI:** 10.1021/acs.joc.2c02080

**Published:** 2022-11-02

**Authors:** Adrian Konopko, Grzegorz Litwinienko

**Affiliations:** † Faculty of Chemistry, University of Warsaw, Pasteura 1, Warsaw02-093, Poland; ‡ Polish Academy of Sciences, Nencki Institute of Experimental Biology, Pasteura 3, Warsaw02-093, Poland

## Abstract

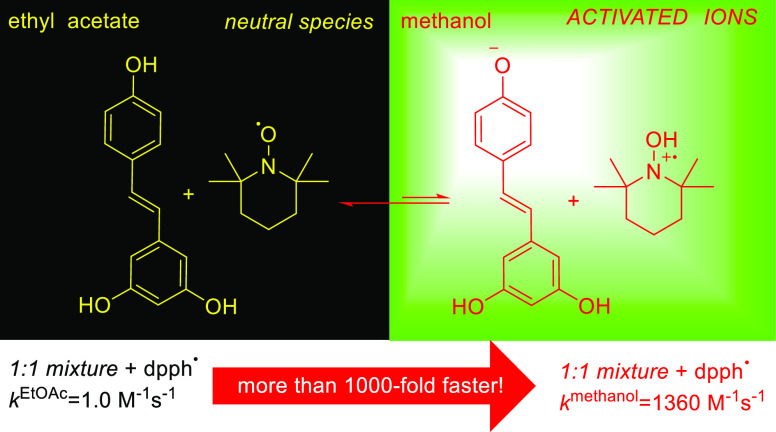

The reaction of the 2,2′-diphenyl-1-picrylhydrazyl radical (dpph^•^) with resveratrol in methanol (*k*^MeOH^ = 192 M^–1^ s^–1^) is greatly accelerated in the presence of stable nitroxyl radical TEMPO^•^ (*k*_mix_^MeOH^ = 1.4 × 10^3^ M^–1^ s^–1^). This synergistic effect is surprising because TEMPO^•^ alone reacts with dpph^•^ relatively slowly (*k*^S^ = 31 M^–1^ s^–1^ in methanol and 0.03 M^–1^ s^–1^ in nonpolar ethyl acetate). We propose a putative mechanism in which a mutual activation occurs within the acid–base pair TEMPO^•^/RSV to the resveratrol (RSV) anion and TEMPOH^•+^ radical cation, both being extremely fast scavengers of the dpph^•^ radical. The fast initial reaction is followed by a much slower but continuous decay of dpph^•^ because a nitroxyl radical is recovered from the TEMPOnium cation, which is reduced directly by RSV/RSV^–^ to TEMPO^•^ or recovered indirectly via a reaction with methanol, producing TEMPOH subsequently oxidized by dpph^•^ to TEMPO^•^.

## 1 Introduction

**Figure 1:**
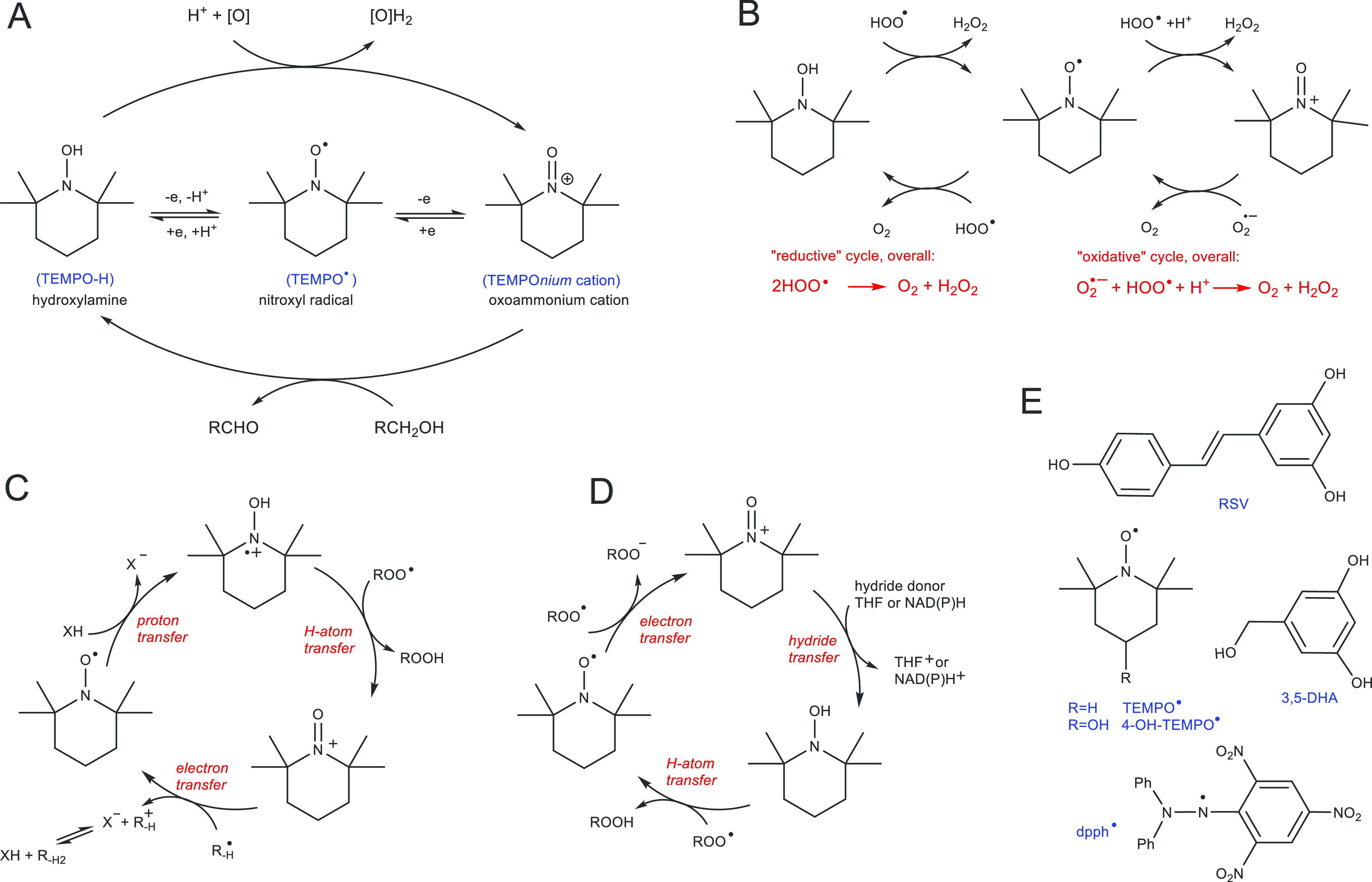
(A) Common redox states of the TEMPO^•^ radical (one electron reduction to hydroxylamine, and one electron oxidation to oxoammonium cation), and the catalytic cycle of alcohol oxidation mediated by the oxoammonium cation (here: TEMPOnium).^2,5^ (B–D) Examples of catalytic cycles in which TEMPO^•^ is involved directly. (B) Reaction of TEMPO^•^ with hydroperoxyl radicals via the oxidative cycle (nitroxyl is oxidized to TEMPOnium cation) in water and acidified organic solvents, and via the reductive cycle (nitroxyl is reduced to hydroxylamine) in organic solvents.^3a^ (C) Acid-promoted activation of TEMPO^•^ to the hydroxylamine radical cation and H-atom transfer to the alkylperoxyl radical, with subsequent electron transfer from the alkyl radical resulting in recovery of TEMPO^•^.^6^ (D) Mechanism of catalytic radical-trapping antioxidant activity of TEMPO^•^ in water and lipid/water systems in the absence of acid.^7^ (E) Investigated compounds: resveratrol (RSV), TEMPO^•^ and 4-OH-TEMPO^•^, 3,5-dihydroxybenzyl alcohol (3,5-DHA), and 2,2′-diphenyl-1-picrylhydrazyl radical (dpph^•^).

Stable 2,2,6,6-tetramethylpiperidine-*N*-oxyl radical (TEMPO^•^, Figure 1) is frequently used as a spin label, radical probe, catalyst for controlled polymerization processes, and mediator of the oxidation of primary and secondary alcohols to the corresponding aldehydes, ketones, and acids.^1,2^ Interestingly, the elementary steps of this catalytic oxidation (i.e., electron or electron/proton transfer) are mediated by the TEMPOH/TEMPOnium^+^ redox couple, without direct participation of the sole nitroxide in the catalytic cycle, see Figure 1A. Other catalytic processes mediated by TEMPO^•^, like dismutation of HOO^•^/O_2_^•–^ to H_2_O_2_ (mimicking superoxide dismutase, SOD), include direct participation of this nitroxyl radical as a necessary component of the catalytic cycle based on the TEMPOnium^+^/TEMPO^•^ pair^3^ (TEMPO^•^ is a reducer), although some examples of TEMPO^•^ acting as an oxidant (involving the nitroxide/hydroxylamine pair) are also reported,^4^ see Figure 1B.

Since the discovery of SOD-like activity of nitroxides,^4^ many biological and therapeutic effects have been associated with their ability to remove reactive oxygen species. The protective effect was associated with a decrease in oxidative stress and the consequent inhibition of oxidation of proteins and lipids, resulting in anti-inflammatory, antioxidant, and antiangiogenic activities. Cyclic nitroxides were found to have protective effects against neurodegenerative diseases (Alzheimer, Huntington, Parkinson), multiple sclerosis, diabetes, ischemia–reperfusion injury, age-related diseases, and many types of cancer (breast, liver, lung, thyroid, ovarian, and lymphatic cancers).^8^ The outstanding ability of TEMPO^•^ to remove superoxide (HOO^•^/O_2_^•–^, see oxidative cycle in Figure 1B) invigorated other mechanistic studies on antioxidant activity and bioactivity of nitroxides. In nonpolar solvents and at ambient temperature, TEMPO^•^ (and other nitroxides) does not react directly with alkylperoxyl radicals^9^ (the main chain carrying species during autoxidation). The antioxidant effect of this cyclic nitroxyl during autoxidation of hydrocarbons at high temperatures is due to a cycle involving the addition of an alkyl radical to TEMPO^•^, subsequent H atom abstraction from alkoxyamine by a peroxyl radical, and β-fragmentation to an aminyl radical, which reacts with another peroxyl radical to regenerate TEMPO^•^.^10^ This mechanism does not work at lower temperatures, thus, nitroxides must be converted into good radical trapping antioxidants (RTA). For example, protonation of the nitroxyl moiety facilitates a fast H-atom transfer from TEMPOH^•+^ to an alkylperoxyl radical, producing a TEMPOnium cation, which is reduced back to nitroxide through electron transfer from an alkyl radical R^•^ (Figure 1C, the reaction is faster than the reaction of R^•^ with O_2_).^6^ Another example of activation was documented in nonpolar systems where peroxidation is mediated by mixed alkyl peroxyl/hydroperoxyl radicals or in the presence of molecules capable of transferring the chain carrying radicals from ROO^•^ to HOO^•^. The inhibition is realized within a cycle of TEMPO^•^ reacting with HOO^•^ (*k*_HOO•_ = 1.1 × 10^9^ M^–1^ s^–1^, Figure 1B, reductive cycle), and the resulting TEMPOH reacts with ROO^•^ (*k*_ROO•_ = 5 × 10^7^ M^–1^ s^–1^).^3a,11^ In this way, reduction of alkylperoxyl radicals is a side effect of the reaction of TEMPO with hydroperoxyl radicals.

Goldstein and Samuni^12^ reported that nitroxides react easily with alkylperoxyl radicals in water (*k*_ROO•_ ≈ 10^7^ M^–1^ s^–1^, with a 5-fold decrease upon increasing the pH from 6.9 to 8.0), and they considered two mechanisms, electron transfer from nitroxide to ROO^•^ or formation of unstable trioxide: >NOOOR, decomposing to >N=O^+^ and ROO^–^ in the presence of acids. Pratt and coworkers^7^ proposed another catalytic cycle (Figure 1D) as an explanation of the inhibiting effect of nitroxyls during THF peroxidation in water and also during oxidation of lipids dispersed in water, with regeneration of the nitroxide as a two-step process that involves transfer of hydride from the substrate to the nitroxide-derived oxoammonium ion followed by H-atom transfer from the resultant hydroxylamine to the peroxyl radical.

The rate constants presented above for the reaction with alkylperoxyls are much higher than *k*_ROO•_ for α-tocopherol (or for Trolox, its water soluble analog) measured under the same conditions. Moreover, the redox cycles, in which nitroxyls are recovered tens or hundreds of times, make them extremely efficient antioxidants. Nitroxyls are cell-permeable (including Brain–Blood Barrier), nontoxic, nonimmunogenic,^8h,8i^ and these persistent radicals are becoming front-line antioxidants.^8f,8h^ The catalytic cycles described above indicate the high redox activity of TEMPO^•^, TEMPOH, and TEMPOnium cation, which should interact with other antioxidant molecules, and here, we present the radical trapping activity of a model system based on TEMPO^•^ and resveratrol (3,4′,5-trihydroxystilbene, RSV, Figure 1E), the latter as another emerging molecule of high bioactivity.^13^

## 2 Results and Discussion

To the best of our knowledge, there is no mechanistic study of the role of a solvent during the reaction of TEMPO^•^ with radicals and with phenolic antioxidants. In order to simplify the chemistry, we used a stable radical, 2,2′-diphenyl-1-picrylhydrazyl (dpph^•^, Figure 1E), which is isoelectronic to peroxyls but is ∼3 orders of magnitude less reactive than alkylperoxyl radicals. Although the results of experiments with dpph^•^ carried out in polar solvents cannot be directly extended on the peroxyl radicals,^14^ careful analysis of the kinetics of the second order reaction with the radical-trapping agent, RTA
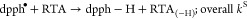
1provides valuable information on the role of polarity, kinetic solvent effects, and antiradical properties of RTAs as putative antioxidants.

### 2.1 Two Different Mechanisms Are Responsible for the Activation of Resveratrol and TEMPO^•^ in Methanol

Reaction 1 was monitored for series of concentrations of RTA being always in stoichiometric excess over the constant initial concentration of dpph^•^. Pseudo-first order experimental rate constants were converted into bimolecular *k*^S^ from the linear least-square slopes derived from the plots of *k*_exp_ versus [RTA], eq 2.^14d,15^
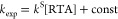
2

The rate constants *k*^S^ , determined for very initial stage of reaction, are collected in Table 1. The rate constant for the RSV/dpph^•^ reaction in ethyl acetate corresponds to HAT/PCET process. This solvent is a good HB acceptor (see footnote b in Table 1), and the reactivity of RSV is greatly diminished because the intermolecular complex ArOH···S is essentially unreactive as explained by the Kinetic Solvent Effect^16^ (the H atom can be abstracted from the “free”, non H-bonded hydroxy group). In contrast to RSV, the nitroxyl moiety in TEMPO^•^ and 4-OH-TEMPO^•^ are not HB donors; thus, the very slow reaction with dpph^•^ in ethyl acetate (see Table 1) must be a consequence of the extremely low intrinsic reactivity of both nitroxides in a nonpolar solvent. When RSV and TEMPO^•^ (or 4-OH-TEMPO^•^) are used together (as equimolar mixture), the rate of the reaction with dpph^•^ is nearly the same (compare *k*^S^’s in Table 1). A similar lack of acceleration in ethyl acetate is also observed for TEMPO-H mixed with RSV.

**Table 1:** Bimolecular Rate Constants, *k*^S^ Calculated from eq 2^a^ for Reactions of dpph^•^ with TEMPO^•^, 4-OH-TEMPO^•^, RSV, 3,5-DHA (See Figure 1E), and Equimolar Mixture of Tested Compounds (1:1, mol/mol) in Methanol (MeOH) and Ethyl Acetate (EtOAc)

RTA	EtOAc^b^	MeOH^b^	MeOH/H^+^
RSV^c^	1.01 ± 0.04	192 ± 7	2.4 ± 0.4
TEMPO^•^	0.03 ± 0.01	31 ± 4	132 ± 2
RSV + TEMPO^•^	0.97 ± 0.13	1360 ± 260	
4-OH-TEMPO^•^	0.07 ± 0.03	12.6 ± 1.7	15.6 ± 3.8
RSV +4-OH-TEMPO^•^	1.21 ± 0.04	580 ± 16	
3,5-DHA^c^		0.63 ± 0.06	
3,5-DHA + TEMPO^•^		107 ± 1	
TEMPO-H	7.7 ± 1.7	35 ± 1	44 ± 3
RSV+ TEMPO-H	9.0 ± 1.7	2100 ± 100	

a Full statistical and kinetic data, together with plots of eq 2 are given in the Supporting Information. When a 1:1 mixture was used, *k*^S^ was calculated from eq 2 with the assumption that [RTA] = [RSV] because for the initial rates RSV is a kinetically dominating RTA, see explanation in the text.

b HB accepting ability expressed as β_2_^H^ parameter is 0.41 for methanol and 0.45 for EtOAc. Methanol is also a donor for HB, its parameter of HB acidity, α_2_^H^ = 0.367.^17^ The relative permittivities of methanol and ethyl acetate are 32.7 and 6.0, respectively.

c p*K*_a_ values for RSV: 9.0 (for the 4’OH group), 9.8 and 11.3 (for the 3 and 5 OH group), see the discussion in ref (18); p*K*_a_ predicted for 3,5-DHA is 9.4.^18^

When passing from ethyl acetate to methanol, the reactivity of each compound greatly increases;^19,20^ there is a 200-fold increase of *k*^S^ for RSV and for 4-OH-TEMPO^•^, and that for TEMPO^•^*k*^MeOH^ is three orders of magnitude bigger than *k*^EtOAc^ (however, much smaller, 4-fold acceleration is observed for TEMPO-H, see Table 1). The large acceleration for RSV cannot be justified as an effect of increased fraction of non-H bonded RSV in methanol compared to ethyl acetate because the ratio *k*^MeOH^/*k*^EtOAc^ calculated from empirical equation^21^ for Δβ_2_^H^ = 0.04 (see footnote *b* in Table 1) could not be greater than 2. Therefore, acceleration observed for RSV in methanol can be assigned to another mechanism, the Sequential Proton-Loss Electron Transfer (SPLET), which is typical for dpph^•^ reacting with phenols in polar, ionization supporting solvents like water and alcohols.^14d,15,16b,22^ SPLET includes three steps: (i) ionization of a phenol and (ii) electron transfer from phenoxide anion to the dpph^•^ radical to form dpph^–^, which undergoes protonation (iii) to give dpph-H. The addition of acetic acid to methanol reduces the reaction rate exactly to the level for the pure HAT process, and *k*^MeOH^ ≈ 2*k*^EtOAc^, as predicted for Δβ_2_^H^ = 0.04, see Table 1 and ref (21).

Obviously, the above explanation is valid for RSV but fails for nitroxyl radicals because they are not HB donors, and the SPLET mechanism does not exist for the nitroxyl moiety. However, autoprotolysis of methanol could be a gentle source of protons interacting with nitroxyls, and the acceleration of reaction observed by TEMPO^•^ and 4-OH-TEMPO^•^ can be attributed to protonation of the minute fraction of nitroxyls. Such traces of TEMPOH^•+^ are sufficient to effectively accelerate the process. The experiment with 10 mM acetic acid added to methanol confirms this hypothesis because an additional 4-fold increase in *k*^S^ is observed (Table 1), in full agreement with the mechanism of the acid-promoted reaction of TEMPO^•^ with peroxyl radicals,^6^ see Figure 1C. Here, we confirmed this mechanism for dpph^•^. It should be noted that acetic acid does not substantially accelerate the rate of the reaction of dpph^•^ with 4-OH-TEMPO^•^, but introduction of the 4-hydroxy group might enhance the acidity of the nitroxyl moiety (by analogy, 4-OH-TEMPOH_2_^+^ is much more acidic than TEMPOH_2_^+^, see Table 2, and see also ref (38) with indicated difference in acidity of TEMPO^•^ and 4-OH-TEMPO^•^). Protonation/deprotonation of the X functional group in 4-X-TEMPO^•^ is also important for redox properties (*E*^0^ and Bond Dissociation Enthalpy, BDE), for example, the BDE (O–H) in 4-X-TEMPO-H is 5.7 kcal/mol higher for X = NH_3_^+^ than for X = NH_2_.^5^ In our system, the expected gain in reactivity after addition of acetic acid is lost because 4-OH_2_^+^-TEMPO^•^ is less active than 4-OH-TEMPO^•^.

**Table 2:** Acidity Parameters and O–H Bond Dissociation Enthalpy (BDE) for Nitroxides, Their Derivatives, RSV, and Reduced dpph^•^

	p*K*_a_	BDE kcal/mol
TEMPOH^•+^^a^	–5.5 ± 1,^26^–5.8 ± 0.3^27^	∼70^7^
4-OH-TEMPOH^•+^	–6.2^27^	
TEMPOH	12.9^b^	(69–72.6)^5,28^ 70.4^c^, 66.5, 66^d^
4-OH-TEMPOH		70.9^c^
TEMPOH_2_^+^	7.36,^1,5^ 7.96,^29^ 7.5,^e^ 6.90^27,30^	116^7^
4-OH-TEMPOH_2_^+^	5.18,^29^ (6.9–7.1),^31^(6.0 ± 0.3)^30a^	
RSV	∼9^f^	83.7^g^
dpph-H	8.54, 8.59^h^	78.9 (N–H)^32^

a Both values determined in H_2_SO_4_. In ref (26), the authors observed that TEMPO is protonated in a system with Hammet H_0_ values more negative than −7.5 (80% H_2_SO_4_) and unprotonated signal for H_0_ > −3.7 (54% H_2_SO_4_), and “between these two acidities, no epr signal is observed because of line broadening resulting from rapid proton exchange”.

b For diethylhydroxylamine in water.^28^

c Calculated from the thermodynamic cycle, from the redox potential, the comproportionation equilibria, and p*K*_a_ of the protonated nitroxyl radical.^30^

d TEMPO-H bond dissociation free energy in acetonitrile calculated from the thermochemical cycle to convert the standard potential to BDFE.^28^

e Determined electrochemically and kinetically.^31^

f The literature values of p*K*_a1_ for RSV are within the range of 6.4–9.7, with the most reliable value ca. 9, see discussion in ref (18).

g BDE for the weakest O–H bond in RSV are discussed elsewhere; the values are scattered from 75.3 to 88.5 kcal/mol.^18^

h In water / methanol (1:1).^33^

### 2.2 Synergy of TEMPO^•^ and RSV

The most surprising effect was observed for dpph^•^ reacting with equimolar mixture of RSV and nitroxide. In contrast to *k*^S^ = 0.97 M^–1^ s^–1^ for the reaction in ethyl acetate, which does not vary from *k*^S^ obtained for the RSV and TEMPO^•^ used separately, the mixture of RSV/TEMPO^•^ in methanol reacts at least 6 times faster than the compounds used independently. When *k*^MeOH^ is compared to *k*^EtOAc^, a big 1400-fold acceleration is observed for RSV/TEMPO^•^, see Table 1. A high 480-fold acceleration is also observed for RSV/4-OH-TEMPO^•^. This surprising acceleration was also observed when RSV was replaced with 3,5-DHA, i.e., for a phenol being 50-fold less reactive (toward dpph^•^) than TEMPO^•^, the mixture of 3,5-DHA with TEMPO^•^ reacted much faster than these two compounds used separately, see Table 1, confirming that such a “synergy” exists for other phenols.

The accessible literature survey indicates that the interactions of the nitroxyl group with phenols in aprotic solvents are limited to the formation of the H bond, quantitatively determined by NMR, FTIR, and EPR measurements^23^ (TEMPO^•^ is a hydrogen bond acceptor, with β_2_^H^ = 0.46^23b^). The reaction of nitroxyls with some of the most reactive phenolic RTAs in methanol is very slow.^24,25^ To be sure that no reaction occur between RSV and TEMPO^•^, we monitored the absorbance of TEMPO^•^ (at 440 nm) mixed with RSV in methanol, and no change was recorded within 2 h.

Another explanation for the enhanced antiradical reactivity of nitroxyl mixed with RSV is an acid–base equilibrium, which is immediately established after mixing both compounds in methanol, reaction 3. Due to large differences in p*K*_a_ for RSV and TEMPOH^•+^ (Table 2), the equilibrium is strongly shifted to the left; however, even traces of the products are kinetically significant, as previously described for phenols reacting via the SPLET mechanism;^14d,15,16b,22^ thus, electron transfer (reaction 4) would be responsible for a great acceleration of the reaction rate.
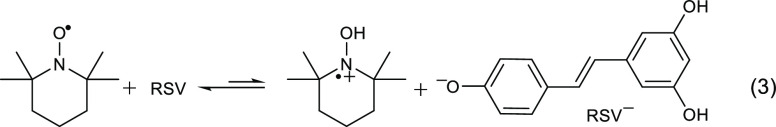
3
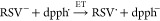
4

The most intriguing is that reaction 3 activates both reacting species, RSV and TEMPO^•^, because TEMPOH^•+^ is extremely good RTA, with BDE O–H = 70 kcal/mol (Table 2) being approximately 8 kcal/mol lower than for O–H in α-tocopherol (the rate constant for the reaction of TEMPOH^•+^ with alkylperoxyl radicals in acetonitrile in the presence of 10 mM *p*-toluenesulfonic acid is ∼1 × 10^8^ M^–1^ s^–1^, i.e., two orders of magnitude higher than *k* = 6.8 × 10^5^ M^–1^ s^–1^ for α-tocopherol).^6a^ Thus, apart from reaction 4, reaction 5 would be an alternative way for decay of dpph^•^reaction 5 would be an alternative way for decay of dpph^•^, with TEMPOH^•+^ reacting via HAT or PCET.
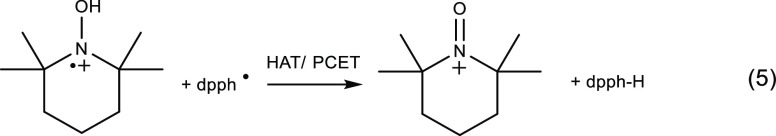
5

Reaction 5 should be slower than reaction 4 (fast electron transfer), but a question appears about the kinetic contribution of reaction 5 to the overall 7-fold acceleration of reaction for the RSV/TEMPO mixture. For TEMPO^•^ (alone) reacting with dpph^•^ in methanol, the presence of 10 mM acetic acid causes 4-fold acceleration, but more reliable contribution of protonated nitroxyl can be estimated when a weaker acid was used, like hexafluoroisopropanol (HFIP) with p*K*_a_ = 9.3 comparable to the acidity of RSV. The *k*^S^ = 61.3 ± 2.1 M^–1^ s^–1^ obtained in the presence of 10 mM HFIP is bigger than *k*^S^ for TEMPO^•^ in neat methanol. Assuming that reaction 3 produces the same amount of TEMPOH^•+^ and RSV^–^, and neglecting HAT from nonionized RSV to dpph^•^ (as the rate in ethyl acetate is very small compared to the overall rate in methanol), we estimated the contribution of HAT from TEMPOH^•+^ to dpph^•^ as 5% (61 M^–1^ s^–1^/1320 M^–1^ s^–1^), and the remaining 95% is due to ET from RSV^–^ to dpph^•^.^34^

Reaction 3 produces two species that hypothetically could react via ET in reaction 6.
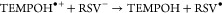
6

Aliaga et al.^24b^ estimated the rate of reaction 6 as diffusion controlled (or even greater due to Coulombic attraction and proximity of ions formed in reaction 3). From a formal point of view, the sequence of reactions 3 and 6 gives a two-step H atom transfer (H^+^, e^–^) from RSV to TEMPO^•^; that is not the case because, as described above, TEMPO^•^ does not react with RSV. Moreover, if reaction 6 was significant, TEMPOH instead of RSV^–^ would be the main scavenger of dpph^•^ and the observed overall *k*^S^ determined for the equimolar mixture would be compared to *k*^S^ obtained for the reaction of dpph^•^ with TEMPOH used alone:
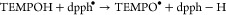
7

However, *k*_7_ = 35 M^–1^ s^–1^ in methanol (see Table 1) is approximately 40-fold smaller than 1360 M^–1^ s^–1^ for the equimolar TEMPO^•^/RSV mixture; therefore, we can exclude reaction 7 from our kinetic considerations. There is also another proof that TEMPOH is not present at the initial steps of reaction because formation of TEMPOH in the vicinity of RSV would generate a new acid–base equilibria, eq 8, producing reactive RSV anions and accelerating the reaction with dpph^•^.
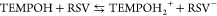
8

The kinetic consequences of reaction 8 were demonstrated in a separate experiment, with TEMPOH introduced to the system (equimolar mixture with RSV), and the reaction was even faster than for RSV/TEMPO^•^, as can be seen in Table 1. Such an acceleration observed for the RSV/TEMPOH acid/base pair can be assigned to the SPLET mechanism in the presence of an RSV anion generated in reaction 8, not to HAT from TEMPOH_2_^+^ to dpph^•^, because BDE_O–H_ for TEMPOH_2_^+^ is much higher than for TEMPOH, see Table 2. There is apparently a counterintuitive acceleration of the reaction of TEMPOH in acidified methanol (reaction is 25% faster than in non-acidified methanol, see Table 1). We explain this surprising acceleration as an effect of the reaction of a nonprotonated fraction of TEMPOH with dpph^•^ (reaction 7), generating a small amount of TEMPO^•^ subsequently protonated by acetic acid and then quickly reacting with dpph^•^ (reaction 5).

### 2.3 Bimodal Kinetics and Continuous Depletion of dpph^•^

**Figure 2:**
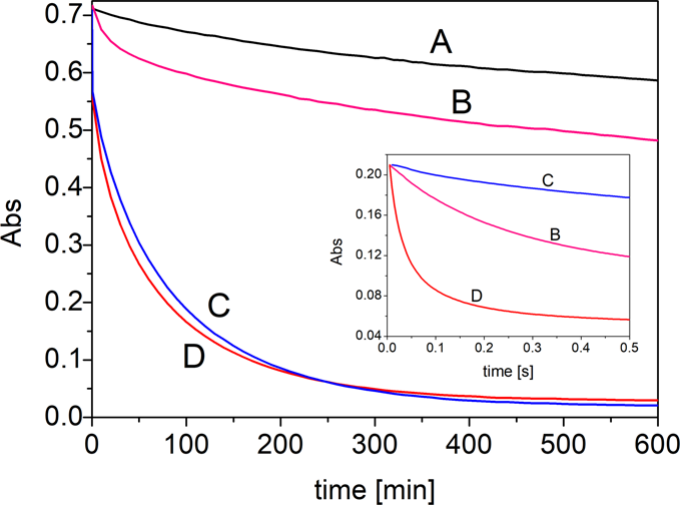
Absorbance of dpph^•^ in methanol monitored during 10 h at 517 nm at ambient temperature: self-decay of dpph^•^ (line A) and mixed with RSV (line B), TEMPO^•^ (line C), and RSV/TEMPO^•^ (line D). The initial concentration of dpph^•^ was always 79 μM (corresponding to Abs = 0.716 in each experiment), and the initial (individual) concentrations of RSV and TEMPO^•^ were 15.8 μM. Reactions were carried out in quartz cuvettes (optical path length = 10 mm), with manual mixing of reagents. Inset: kinetic traces recorded by the stopped flow technique (optical path length = 2 mm) during the first 500 ms of dpph^•^ (86 μM) reacting with 12.4 mM RSV (line B), 12.7 mM TEMPO^•^ (line C), and with an equimolar mixture (10.4 mM TEMPO^•^ + 10.4 mM RSV, line D). Please note the inversion of the rates of the process: the fast reaction of RSV with dpph^•^ at the initial step is becoming much slower when observed for a time longer than 1 min. See the discussion in the text about changing kinetics.

In order to check the stoichiometry of the reaction, we monitored absorbance of dpph^•^ (5-fold stoichiometric excess) reacting with RSV, TEMPO^•^, or a mixture of RSV/TEMPO^•^ in methanol during 10 h (Figure 2). Control experiments with dpph^•^ alone indicated a slow self-decay (17% during 10 h). For 79 μM dpph^•^ reacting with 15.8 μM RSV, about 30% of the radical was consumed (Figure 2, line B). Curve B in Figure 2 demonstrates a rather low conversion of dpph^•^ during the reaction with RSV (dpph^•^ is in 5-fold excess), and it can be concluded that one molecule of RSV does not trap more than two radicals. This observation (for dpph^•^/RSV) is in agreement with the rather low stoichiometric factor (*n* = 1.9) determined by Amorati et al.^35^ for RSV trapping peroxyl radicals in chlorobenzene, and is much lower than *n* ≈ 5 recently observed by us^18^ for RSV during peroxidation carried out in heterogeneous systems and explained to be due to dimerization of RSV^•^ in micelles or liposomes, where the local concentration of RSV^•^ is much higher than in a continuous phase. For the same reasons we exclude dimerization or any other activation/regeneration of RSV in methanol. Formal H atom transfer from RSV to dpph^•^ (reaction 9) is endothermic and reversible (BDE_N–H_ in dpph-H is lower than BDE_O–H_ in RSV, see Table 2), with a relatively slow shift toward products due to irreversible quenching of RSV^•^ (Figure 2, line B).
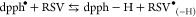
9

The results for dpph^•^ reacting with TEMPO^•^ (used alone or mixed with RSV, Figure 2, lines C and D) are quite surprising because continuous depletion was observed. For TEMPO^•^ reacting alone, the half-life for dpph^•^ was 34 min, and after 10 h, 97% of dpph^•^ was consumed (Figure 2, line C). The mixture of RSV with TEMPO^•^ was even more effective, with a half-life of 22 min, and after 10 h, 95% of dpph^•^ was consumed (line D). In contrast to the fast reaction at the beginning (*k*^S^ values were calculated for the first half-second of the reaction using the stopped flow technique), the long lasting monitoring of the dpph^•^ decay suggests that after the initial fast reaction, TEMPO^•^ alone or mixed with RSV follows a different kinetics, with drastically slower but continuous consumption of dpph^•^.

Such bimodal kinetics can be reasoned as a result of slow evolution of intermediates, which immediately react with dpph^•^. TEMPOH^•+^ with its high reduction potential (*E*^0^_red_ = 955 mV for TEMPOH^•+^/TEMPOH)^27,36^ is an efficient oxidizing agent for TEMPO^•^ (*E*^0^_red_ = 750 mV for the TEMPOnium^+^/TEMPO^•^ pair),^27,37^ and the process can be presented as a sequence of reversible reactions:^38,30a^
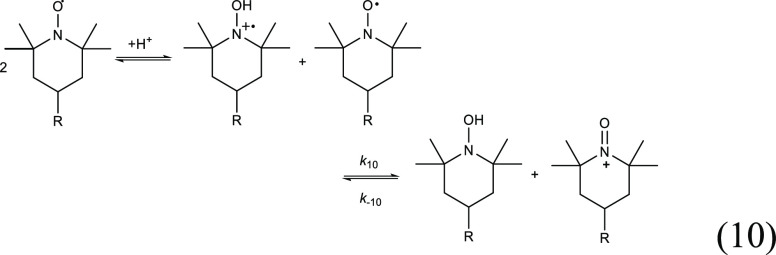
10with the solvent-independent rate constant *k*_10_ = (1.4 ± 0.8) × 10^5^ M^–1^ s^–1^ (in diluted solution).^27^ It is very likely that reaction 10 contributes to more efficient protonation of TEMPO^•^ by RSV because it consumes TEMPOH^•+^; thus, it is an additional driving force for reaction 3, which is shifted to the right).

The reverse process, known as comproportionation, is much slower, with pH-dependent *k*_–10_ , ranging from 0.23 M^–1^ s^–1^ at pH 4.6 to 51 ± 1 M^–1^ s^–1^ at pH 10 (data for TEMPO^•^).^27,31^ The resulting hydroxylamine is known as an efficient inhibitor of peroxidation.^6,7,39^ TEMPOH reacts rapidly with peroxyl radicals, with *k* from 5 × 10^5^ to 3 × 10^6^ M^–1^ s^–1^,^6^ and also should effectively react with dpph^•^ via HAT (BDE_O–H_ in hydroxylamines and BDE_N–H_ for dpph^•^ are listed in Table 2); however, from the kinetic point of view, reaction 10 does not bring any additional acceleration because the reactivity of TEMPOH^•+^ (maximal *k^S^* = 61 M^–1^ s^–1^, vide supra) is similar to the reactivity of TEMPOH (*k^S^* = 35 M^–1^ s^–1^). The main benefit is an increase in overall stoichiometry, regardless reaction 5 or 10 is dominant, since both reactions produce a TEMPOnium cation, which is a strong oxidant capable of abstracting electron from the alkyl radical (*k* ≈ 1–3 × 10^10^ M^–1^ s^–1^, as presented in Figure 1C)^6b,40^ and hydride from THF (*k* ≈ 5.3 × 10^–5^ M^–1^ s^–1^, Figure 1D).^41^ TEMPO^+^ might also oxidize methanol, reaction 11,^3b,12,42^ with *k* = 0.48 ± 0.02 M^–1^ s^–1^ (stopped flow technique^3b^). Reaction 11 is of synthetic importance,^2^ and the mechanism is pH dependent^42,43^ with bimolecular hydride transfer in acidified and in neutral methanol.^43,44^
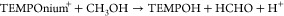
11

Thus, reaction 11 combined with reactions 5 and 10 gives a slow flux of TEMPOH as a radical trapping agent during a prolonged reaction (when the initial fast reaction is over). The TEMPOnium cation could also be reduced by RSV via HAT, or even by ET (reaction 12), giving recovered TEMPO^•^.
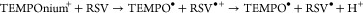
12

Please note that both reactions 11 and 12 produce a proton, which can facilitate a disproportionation of TEMPO^•^ to TEMPOH and TEMPOnium cation (reaction 10). On the other hand, TEMPOH generated in reaction 11 is a base (see Table 2,) which might produce an additional portion of RSV anions, see eq 8. An excess of neutral RSV should still be present in the system because of the endothermic and reversible character of reaction 9 (Figure 2, line B). Another way for the reduction of TEMPOnium^+^ to TEMPO^•^ is the reaction with deprotonated the resveratryl radical, which is also present in the system. In our previous publication^18^ we estimated p*K*_a_ for RSV^•^ as between 6.5 and 7.0 (in water), based on the reports by Kerzig et al.^45^ The same researchers determined the half-life for (RSV^•^/RSV^•–^, H^+^) as 50 μs; thus, the reaction of radical anions with TEMPOnium cations cannot be excluded, and the Coulombic attractions of the reacting species will further accelerate this process. The increased acidity of RSV^•^ compared to RSV also has an additional consequence for the initial kinetics of dpph^•^ decay because a new acid/base equilibria is established, with an additional amount of TEMPOH^•+^ generated in the system.

Basing on the redox potentials collected in Table S22, a reaction of TEMPOnium^+^ with dpph^–^ could be also considered; however, the kinetic significance of such a reversible process is rather low because [dpph^–^] ≪ [RSV] ≪ [methanol]; thus, the reaction of TEMPOnium^+^ with RSV and methanol will be preferred as possible ways of recovery of TEMPO^•^. Moreover, the reaction of dpph^–^ with TEMPOnium^+^ would regenerate dpph^•^, but inspection of Figure 2 indicates that TEMPO^•^ used alone or mixed with RSV is a very efficient radical trapping agent. An anonymous reviewer suggested the additional (and not excluding) explanation for the increased number of dpph^•^ radicals trapped by RSV mixed with TEMPO^•^ (comparing to RSV used alone) during extended reaction time (Figure 2) as an effect of the reaction of TEMPO^•^ with RSV^•^, being an additional driving force for the reversible reaction 9.

### 2.4 General Scheme for the Cooperation of TEMPO^•^ with RSV

**Scheme 1:**
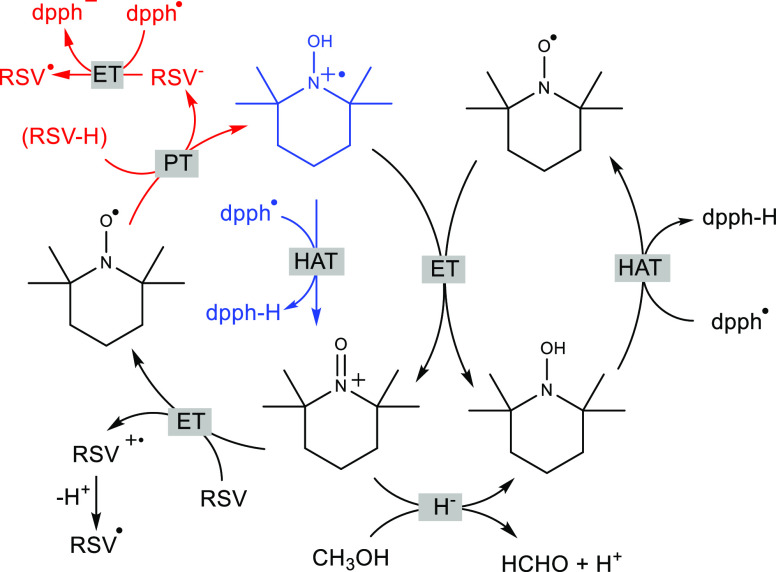
Mechanism of the Reaction of dpph^•^ with Resveratrol and TEMPO^•^ in Methanol The initial step (fast reaction of dpph^•^ with RSV anions, is marked in red, and fast reaction of TEMPOH^•+^ with dpph^•^, marked in blue) is followed by slow kinetics resulting from the cyclic reactions including protonation of TEMPO^•^, reaction of oxoammonium cation with dpph^•^ and/or comproportionation, formation of TEMPOnium cation and TEMPOH, and regeneration of TEMPO^•^ (see the main text).

Scheme 1 presents reactions 3–5 and 7–8 assembled into a mechanism that explains the observed bimodal kinetics. Both compounds form an acid/base pair with the activated species: resveratrol anion and hydroxylamine radical cation. Fast bleaching during the very initial step of the reaction is mainly a consequence of two processes: electron transfer from the resveratrol anion to dpph^•^ (as predicted by the Sequential Proton-Less Electron Transfer mechanism, marked in red) and fast HAT from the hydroxylamine radical cation to dpph^•^ (marked in blue). The much slower decay of dpph^•^ during the next several hours was caused by HAT from hydroxylamine formed in at least two reactions (10 and 11) and by slow recovery of TEMPO^•^. Note that the RSV^–^ anion after the reaction with dpph^•^ is converted to RSV^•^, which is acidic and might also serve as an acid in the Brönsted acid/base pair with TEMPO^•^.

Despite many limitations of dpph^•^ radical mentioned in several works,^14^ this radical is still considered as a useful and convenient model for peroxyl radicals and is widely used for prescreening of putative RTAs. One of the main objections against the application of this electron-deficient radical is that in polar solvents, phenols react with dpph^•^ via the mixed HAT and ET (SPLET) mechanism, which is not relevant to the nonpolar environment in hydrocarbons/polymers/biomembranes. However, recently observed reactivity of nitroxides as inhibitors of peroxidation in solutions (THF in water) and in dispersed systems (lipid bilayers and ferroptosis)^7^ revealed participation of HAT, PT, ET, and even hydride transfer (Figure 1D and reaction 11 as a part of Scheme 1) as key steps of the catalytic turnover of the nitroxide. If a good hydride donor is not available in the lipid phase, the generated oxoammonium cations diffuse to the lipid/water interface where they might be reduced by water-soluble reductants (e.g., by NADPH, ascorbate, etc.). Our experiments, although carried out with dpph^•^ radical instead of peroxyl radicals, demonstrate that phenols (water or lipid soluble) might play a significant role in the recovery of TEMPO^•^.

## 3 Conclusions

In methanol, dpph^•^ reacts six times faster with RSV than with TEMPO^•^; however, when both compounds are used together as an equimolar mixture, the rate constant for the reaction with dpph^•^ is one order of magnitude bigger than for any of the components used alone. Acceleration is caused by RSV anions and TEMPOH^•+^, and both species are much more reactive than their neutral parent compounds. Although acid promoted activation of TEMPO^•^ was previously reported,^6^ our observation is particularly interesting as an example of a dramatic kinetic consequence of very subtle acid base equilibria because TEMPO^•^ is an extremely weak base and the difference in p*K*_a_ between TEMPOH^•+^ and RSV is about 15 units. Moreover, the observed acceleration is an effect of a mutual activation of RSV and TEMPO^•^, with 95% of the dpph^•^ reduced by RSV anions via ET and ca. 5% of dpph^•^ reacts with TEMPOH^•+^ via the HAT process.

Apart from relatively simple kinetics observed for very initial reaction rates, the reaction monitored during extended time (10 h) reveals a slow, continuous decay of dpph^•^ (used in 5-fold excess over TEMPO^•^ and RSV), resulting in the stoichiometry exceeding the number of radicals trapped by typical phenolic antioxidants. Based on the result for TEMPO^•^ reacting with peroxyl radicals published previously by Pratt and coworkers,^6,7^ we propose a putative cyclic mechanism of oxidation and reduction of TEMPO^•^ (Scheme 1) including the role of methanol and RSV as reducing agents for the oxoammonium cation, facilitating the recovery of TEMPO^•^ and providing a continuous flux of activated TEMPO^•^ derivatives being efficient radical trapping agents (Scheme 1). We expect such mutual activation of phenols and nitroxyls to be conserved among many ArOH/TEMPO^•^ pairs reacting with ROO^•^ in biological systems. Cooperation of phenols with nitroxyls might be of practical importance for the design and synthesis of hybrid compounds with nitroxyl and phenol functionalities, resulting in materials and biomaterials with improved antiradical activity, as recently proposed for polydopamine nanoparticles decorated with nitroxyls.^46^

## 4 Experimental Section

Commercially available *trans*-RSV, TEMPO^•^, dpph^•^, 4-OH-TEMPO^•^, TEMPOH, and 3,5-DHA were used as received (3,5-DHA was recrystallized from methanol before use).

The results of kinetic studies on solvent effects, acid–base catalysis, and reaction mechanism are extremely sensitive on any impurities,^22^ and our recent work with catecholamines^15^ indicates straightforward variation of *k*^S^ on pH.^47^ Since even traces of base present in a solvent distilled over CaH_2_ are kinetically significant,^22^ methanol and ethyl acetate were distilled over a few crystals of dpph^•^ and a few beads of amberlite resin in order to remove traces of stabilizers and alkaline impurities. Before each series of experiments, the kinetic purity of methanol was tested by comparison of the rate constant obtained for 2,4,6-trimethylphenol (reference phenol) with a reference value,^22^ i.e., *k*^S^ = (43 ± 15) M^–1^ s^–1^.

Stopped-flow measurements were made following the procedure described elsewhere.^14d,15^ The decay of dpph^•^ (concentration between 6 and 9 × 10^–5^ M) in the stoichiometric excess of the tested compounds (concentration between 1 and 2 × 10^–2^ M) was monitored at 517 nm on an Applied Photophysics SX 20 stopped-flow apparatus, equipped with a xenon arc lamp source and photodiode array detector. The mixing cell (2 mm optical path length, dead-time of mixing was 1.1 ms) and the tubes with the reactants were thermostated at 25 °C. Measurements were performed in neat methanol, ethyl acetate, and acidified solvents (10 mM acetic acid or 10 mM hexafluoroisopropanol). The initial rates of reaction were monitored, giving the pseudo-first-order rate constant (*k*_exp_), calculated as the average from at least two sets of measurements. The plots of the *k*_exp_ vs concentration the tested compounds were linear, and their slopes gave the second-order rate constants, *k*^s^.

Extended (ten hours) experiments of the decay of dpph^•^ were monitored with a Varian Cary 50 spectrometer and QS quartz cuvettes with a 10 mm optical path length. A total of 100 μL of dpph^•^ (final concentration: 79 μM) in methanol and 30 μL of TEMPO^•^, RSV or TEMPO^•^, and RSV (final concentration: 15.8 μM) were injected to 3 mL of methanol in a quartz cuvette. Absorbance data was collected every 10 min (517 nm).

## Supporting Information Available

Kinetic data for the reaction of TEMPO^•^, 4-OH-TEMPO^•^, RSV, 3,5-DHA and the equimolar mixture of TEMPO^•^/RSV, 4-OH-TEMPO^•^/RSV, and TEMPO^•^/3,5-DHA with dpph^•^ in ethyl acetate, methanol, and acidified methanol; and literature values of oxidation and reduction potentials for TEMPO^•^, 4-OH-TEMPO^•^, TEMPOH^•+^, TEMPOnium^+^, RSV, and dpph^•^ (PDF)
