# Supplementary material for: Mutual Activation of Two Radical Trapping Agents: Unusual “Win–Win Synergy” of Resveratrol and TEMPO during Scavenging of dpph• Radical in Methanol
Source: J Org Chem. 2022 Nov 2;87(22):15530–8. doi: 10.1021/acs.joc.2c02080 (PMC9680031; doi:10.1021/acs.joc.2c02080)
Supplement: Supplementary file 1 — jo2c02080_si_001.pdf [file jo2c02080_si_001.pdf]

Supporting Information

Mutual activation of two radical trapping agents: unusual "win-win synergy" of resveratrol and TEMPO during scavenging of dpph$^•$ radical in methanol.

Adrian Konopko, [1,2] **& Grzegorz Litwinienko**[1,*]

[1] *Faculty of Chemistry, University of Warsaw, Pasteura 1, 02-093, Warsaw, Poland*
[2] *Nencki Institute of Experimental Biology, Polish Academy of Sciences, Pasteura 3, Warsaw, 02-093, Poland*

*Corresponding author (GL) e-mail: litwin@chem.uw.edu.pl

SYMBOLS and ABBREVIATIONS

dpph$^•$	2,2-diphenyl-1-picrylhydrazyl radical
TEMPO$^•$	2,2,6,6-tetramethylpiperidine-1-oxyl radical
4-OH-TEMPO$^•$ -	hydroxy-2,2,6,6-tetramethylpiperidin-1-oxyl radical
RSV	resveratrol
TEMPO-H	2,2,6,6-tetramethylpiperidin-1-ol
3,5-DHA	3,5-dihydroxybenzyl alcohol
AcOH	acetic acid
MeOH	methanol

TABLE OF CONTENTS

	Title	page
**Figure S1. Table S1**	Data and kinetic parameters for reaction of dpph$^•$ with excess of TEMPO$^•$ in MeOH, linear fitting.	S-3
**Figure S2. Table S2.**	Data and kinetic parameters for reaction of dpph$^•$ with excess of 4-OH-TEMPO$^•$ in MeOH, linear fitting.	S-4
**Figure S3. Table S3.**	Data and kinetic parameters for reaction of dpph$^•$ with excess RSV in MeOH, linear fitting.	S-5
**Figure S4. Table S4.**	Data and kinetic parameters for reaction of dpph$^•$ with excess of TEMPO$^•$ in EtOAc, linear fitting.	S-6
**Figure S5. Table S5.**	Data and kinetic parameters for reaction of dpph$^•$ with excess of 4-OH-TEMPO$^•$ in EtOAc, linear fitting.	S-7
**Figure S6 Table S6.**	Data and kinetic parameters for reaction of dpph$^•$ with excess of RSV in EtOAc, linear fitting.	S-8
**Figure S7. Table S7.**	Data and kinetic parameters for reaction of dpph$^•$ with excess of RSV and TEMPO$^•$ 1:1 mol/mol in MeOH, linear fitting.	S-9
**Figure S8. Table S8.**	Data and kinetic parameters for reaction of dpph$^•$ with excess of RSV and TEMPO$^•$ 1:1 mol/mol in EtOAc, linear fitting.	S-10

**Figure S9. Table S9.**	Data and kinetic parameters for reaction of dpph$^{\bullet}$ with excess of RSV and 4-OH-TEMPO$^{\bullet}$ 1:1 mol/mol in MeOH, linear fitting.	S-11
**Figure S10. Table S10.**	Data and kinetic parameters for reaction of dpph$^{\bullet}$ with excess of RSV and 4-OH-TEMPO$^{\bullet}$ 1:1 mol/mol in EtOAc, linear fitting.	S-12
**Figure S11. Table S11.**	Data and kinetic parameters for reaction of dpph$^{\bullet}$ with excess 3,5-DHA in MeOH, linear fitting.	S-13
**Figure S12. Table S12.**	Data and kinetic parameters for reaction of dpph$^{\bullet}$ with excess of 3,5-DHA and TEMPO$^{\bullet}$ 1:1 mol/mol in MeOH, linear fitting.	S-14
**Figure S13. Table S13.**	Data and kinetic parameters for reaction of dpph$^{\bullet}$ with excess of TEMPO$^{\bullet}$ in acidified MeOH (10mM AcOH), linear fitting.	S-15
**Figure S14. Table S14.**	Data and kinetic parameters for reaction of dpph$^{\bullet}$ with excess of 4-OH-TEMPO$^{\bullet}$ in acidified MeOH (10mM AcOH), linear fitting.	S-16
**Figure S15. Table S15.**	Data and kinetic parameters for reaction of dpph$^{\bullet}$ with excess of RSV in acidified MeOH (10mM AcOH), linear fitting.	S-17
**Figure S16. Table S16.**	Data and kinetic parameters for reaction of dpph$^{\bullet}$ with excess of TEMPO$^{\bullet}$ with 10 mM HFIP, linear fitting.	S-18
**Figure S17. Table S17.**	Data and kinetic parameters for reaction of dpph$^{\bullet}$ with excess TEMPO-H in MeOH, linear fitting.	S-19
**Figure S18. Table S18.**	Data and kinetic parameters for reaction of dpph$^{\bullet}$ with excess of TEMPO-H in acidified MeOH (10mM AcOH), linear fitting.	S-20
**Figure S19. Table S19.**	Data and kinetic parameters for reaction of dpph$^{\bullet}$ with excess of TEMPO-H in EtOAc, linear fitting.	S-21
**Figure S20. Table S20.**	Data and kinetic parameters for reaction of dpph$^{\bullet}$ with excess of TEMPO-H and RSV 1:1 mol/mol in MeOH, linear fitting.	S-22
**Figure S21. Table S21.**	Data and kinetic parameters for reaction of dpph$^{\bullet}$ with excess of TEMPO-H and RSV 1:1 mol/mol in EtOAc, linear fitting.	S-23
**Table S22.**	Literature values of oxidation and reduction potentials (vs NHE) for TEMPO$^{\bullet}$, 4-OH-TEMPO$^{\bullet}$, TEMPOH$^{\bullet+}$, TEMPOnium^{+}, resveratrol and dpph$^{\bullet}$.	S-24

Table S1. Pseudo-first-order rate constant (k_{exp}) for reaction of dpph$^•$ (constant initial concentration) with excess of TEMPO$^•$ in MeOH and bimolecular rate constant k^S calculated as a slope from linear dependence of k_{exp} plotted against concentration of nitroxide: $k_{exp}=k^S[TEMPO^•] + const$, with regression coefficient (R^2) and error $\Delta k^S_{90\%}$ calculated as standard deviation for confidential level 90%.

**Neat MeOH**		**Neat MeOH**	
[TEMPO$^•$] / mM	k_{exp} / s^{-1}	[TEMPO$^•$] / mM	k_{exp} / s^{-1}
12.7	0.52	12.5	0.38
9.53	0.40	9.36	0.33
5.10	0.28	7.02	0.26
3.65	0.24	3.58	0.19
2.43	0.18	2.39	0.13
1.62	0.14	1.59	0.09
0.97	0.11	0.96	0.07
k^s = 33.28 $M^{-1}s^{-1}$		k^s = 27.89 $M^{-1}s^{-1}$	
$\Delta k^S_{90\%}$ = 4.0		$\Delta k^S_{90\%}$ = 3.9	
R^2 = 0.9904		R^2 = 0.9781	
**k^{MeOH} = 30.6 ± 3.8 $M^{-1}s^{-1}$**			

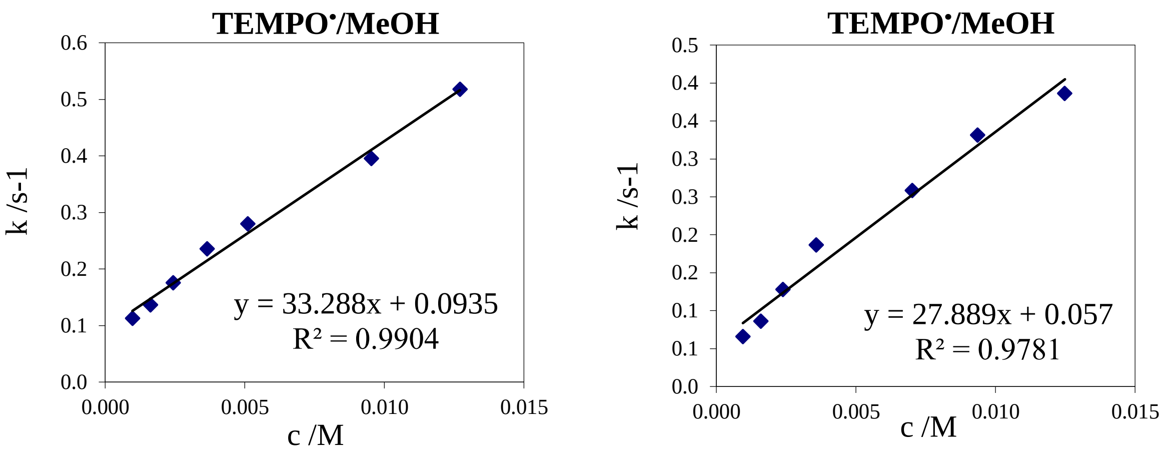

Figure S1. Plots of k_{exp} versus concentration of TEMPO$^•$ (data taken from Table S1).

Table S2. Pseudo-first-order rate constant (k_{exp}) for reaction of dpph$^{\bullet}$ (constant initial concentration) with excess of 4-OH-TEMPO$^{\bullet}$ in MeOH and bimolecular rate constant k^S calculated as a slope from linear dependence of k_{exp} plotted against concentration of nitroxide: $k_{exp}=k^S[\text{4-OH-TEMPO}^{\bullet}]$ + const, with regression coefficient (R^2) and error $\Delta k^S_{90\%}$ calculated as standard deviation for confidential level 90%.

**Neat MeOH**		**Neat MeOH**	
[4-OH-TEMPO$^{\bullet}$] / mM	k_{exp} / s^{-1}	[4-OH-TEMPO$^{\bullet}$] / mM	k_{exp} / s^{-1}
12.6	0.17	9.35	0.13
9.78	0.14	7.01	0.09
7.33	0.13	5.26	0.06
5.50	0.09	2.68	0.03
3.93	0.08	1.79	0.02
2.81	0.06	1.19	0.01
1.87	0.05		
1.25	0.05		
$k^s = 11.4\ M^{-1}s^{-1}$		$k^s = 13.8\ M^{-1}s^{-1}$	
$\Delta k^S_{90\%} = 1.7$		$\Delta k^S_{90\%} = 0.8$	
$R^2 = 0.9839$		$R^2 = 0.9965$	

$k^{MeOH} = 12.6 \pm 1.7\ M^{-1}s^{-1}$

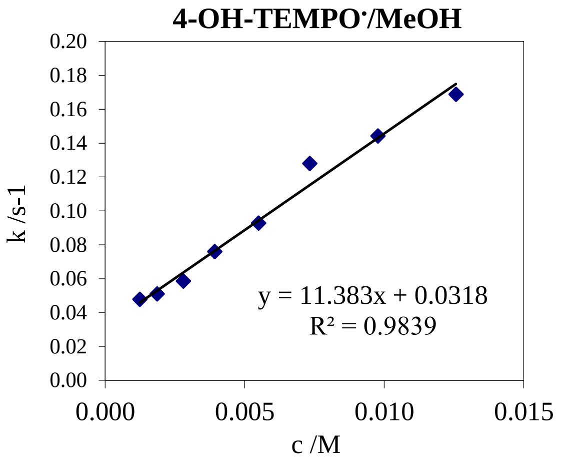

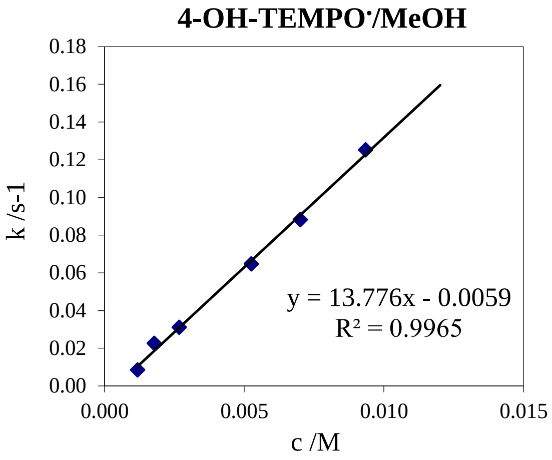

Figure S2. Plots of k_{exp} versus concentration of 4-OH-TEMPO$^{\bullet}$ (data taken from Table S2).

Table S3 Pseudo-first-order rate constant (k_{exp}) for reaction of dpph$^{\bullet}$ (constant initial concentration) with excess of RSV in MeOH and bimolecular rate constant k^S calculated as a slope from linear dependence of k_{exp} plotted against concentration of phenol: $k_{exp}=k^S$[RSV] + const, with regression coefficient (R^2) and error $\Delta k^S_{90\%}$ calculated as standard deviation for confidential level 90%.

Neat MeOH		Neat MeOH	
[RSV] / mM	k_{exp} / s^{-1}	[RSV] / mM	k_{exp} / s^{-1}
12.3	2.77	12.8	2.91
9.56	2.26	9.97	2.47
7.17	1.92	7.48	1.99
5.38	1.53	5.61	1.69
3.84	1.26	4.00	1.41
2.74	0.87	2.86	1.09
1.83	0.74	1.91	0.95
1.22	0.62	1.27	0.71
k^s = 196.24 $M^{-1}s^{-1}$		k^s = 187.06 $M^{-1}s^{-1}$	
$\Delta k^S_{90\%}$ = 16.8		$\Delta k^S_{90\%}$ = 11.3	
R^2 = 0.9914		R^2 = 0.9937	
$\boldsymbol{k^{MeOH}}$ **= 191.7 ± 6.5** $\boldsymbol{M^{-1}s^{-1}}$			

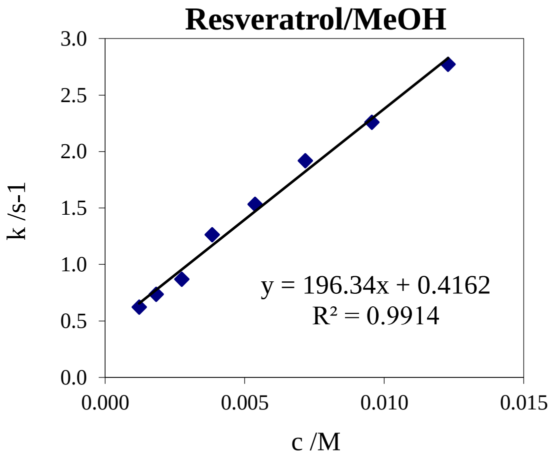

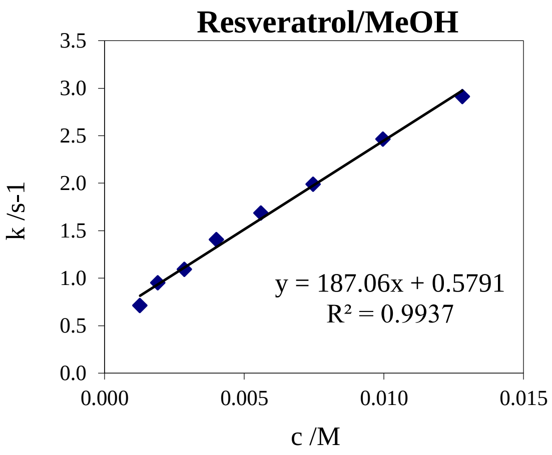

Figure S3. Plots of k_{exp} versus concentration of RSV (data taken from Table S3).

Table S4 Pseudo-first-order rate constant (k_{exp}) for reaction of dpph$^{\bullet}$ (constant initial concentration) with excess of TEMPO$^{\bullet}$ in EtOAc and bimolecular rate constant k^{S} calculated as a slope from linear dependence of k_{exp} plotted against concentration of nitroxide: $k_{exp}=k^{S}[TEMPO^{\bullet}]$ + const, with regression coefficient (R^2) and error $\Delta k^{S}_{90\%}$ calculated as standard deviation for confidential level 90%.

**Neat EtOAc**		**Neat EtOAc**	
[TEMPO$^{\bullet}$] / mM	$k_{exp} \times 10^4$ / s^{-1}	[TEMPO$^{\bullet}$] / mM	$k_{exp} \times 10^4$ / s^{-1}
12.1	6.00	12.2	1.80
9.41	4.73	9.50	1.61
7.06	4.00	7.13	1.21
5.29	3.33	5.35	0.88
3.78	2.75	3.82	0.51
2.70	2.50	2.73	0.39
1.80	2.00	1.82	0.36
1.20	1.47		
k^{s} = 0.039 $M^{-1}s^{-1}$		k^{s} = 0.015 $M^{-1}s^{-1}$	
$\Delta k^{S}_{90\%}$ = 0.003		$\Delta k^{S}_{90\%}$ = 0.002	
R^2 = 0.9913		R^2 = 0.9755	
**K^{EtOAc} = 0.027 ± 0.016 $M^{-1}s^{-1}$**			

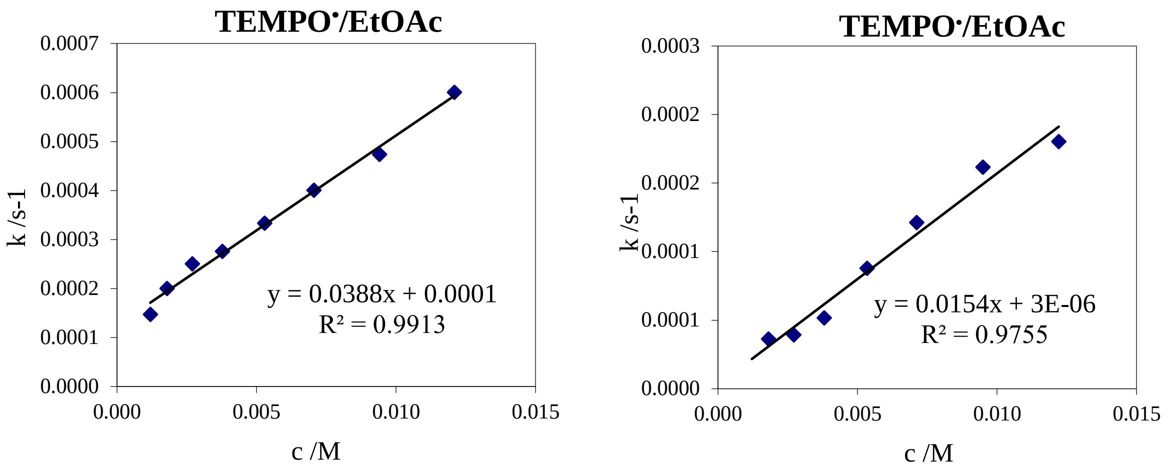

Figure S4. Plots of k_{exp} versus concentration of TEMPO$^{\bullet}$ (data taken from Table S4).

Table S5 Pseudo-first-order rate constant (k_{exp}) for reaction of dpph$^{•}$ (constant initial concentration) with excess of 4-OH-TEMPO$^{•}$ in EtOAc and bimolecular rate constant k^S calculated as a slope from linear dependence of k_{exp} plotted against concentration of nitroxide: $k_{exp}=k^S$[4-OH-TEMPO$^{•}$] + const, with regression coefficient (R^2) and error $\Delta k^S_{90\%}$ calculated as standard deviation for confidential level 90%.

**Neat EtOAc**		**Neat EtOAc**	
[4-OH-TEMPO$^{•}$] / mM	$k_{exp} \times 10^4$ / s^{-1}	[4-OH-TEMPO$^{•}$] / mM	$k_{exp} \times 10^4$ / s^{-1}
11.6	11.0	10.2	6.9
9.04	9.0	7.93	5.4
5.09	5.2	5.95	4.9
3.63	4.0	4.46	4.3
2.60	3.0	3.19	3.9
1.73	2.0	2.28	3.3
1.15	1.0	1.52	2.6
$k^s = 0.094\ M^{-1}s^{-1}$<br>$\Delta k^S_{90\%} = 0.006$<br>$R^2 = 0.9913$		$k^s = 0.045\ M^{-1}s^{-1}$<br>$\Delta k^S_{90\%} = 0.006$<br>$R^2 = 0.9755$	

K^{EtOAc} = 0.07 ± 0.03 $M^{-1}s^{-1}$

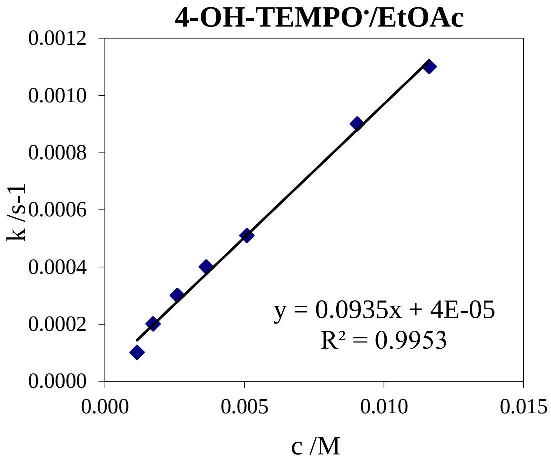

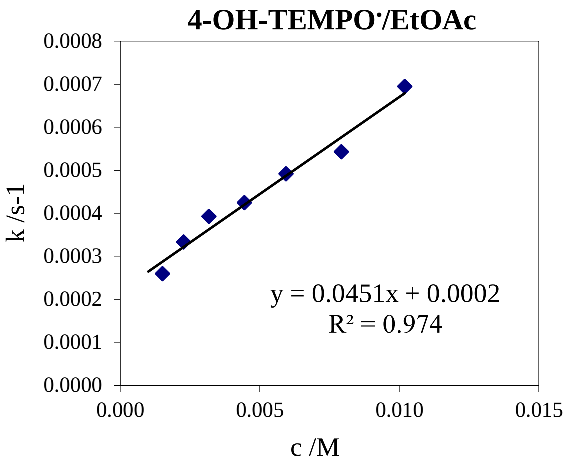

Figure S5. Plots of k_{exp} versus concentration of 4-OH-TEMPO$^{•}$ (data taken from Table S5).

Table S6 Pseudo-first-order rate constant (k_{exp}) for reaction of dpph$^{\bullet}$ (constant initial concentration) with excess of RSV in EtOAc and bimolecular rate constant k^S calculated as a slope from linear dependence of k_{exp} plotted against concentration of phenol: k_{exp}=k^S[RSV] + const, with regression coefficient (R^2) and error $\Delta k^S_{90\%}$ calculated as standard deviation for confidential level 90%.

**Neat EtOAc**		**Neat EtOAc**	
[RSV] / mM	$k_{exp} \times 10^3$ / s^{-1}	[RSV] / mM	$k_{exp} \times 10^3$ / s^{-1}
12.4	12.2	12.7	13.3
9.66	10.0	9.86	10.6
7.24	7.7	7.39	8.1
5.43	5.9	5.55	6.1
3.88	4.4	3.96	4.8
2.77	3.1	2.83	3.3
1.85	2.1	1.89	2.2
1.23	1.4	1.26	1.6
k^s = 0.97 $M^{-1}s^{-1}$		k^s = 1.03 $M^{-1}s^{-1}$	
$\Delta k^S_{90\%}$ = 0.04		$\Delta k^S_{90\%}$ = 0.03	
R^2 = 0.997		R^2 = 0.9986	
**K^{EtOAc} = 1.00 ± 0.04 $M^{-1}s^{-1}$**			

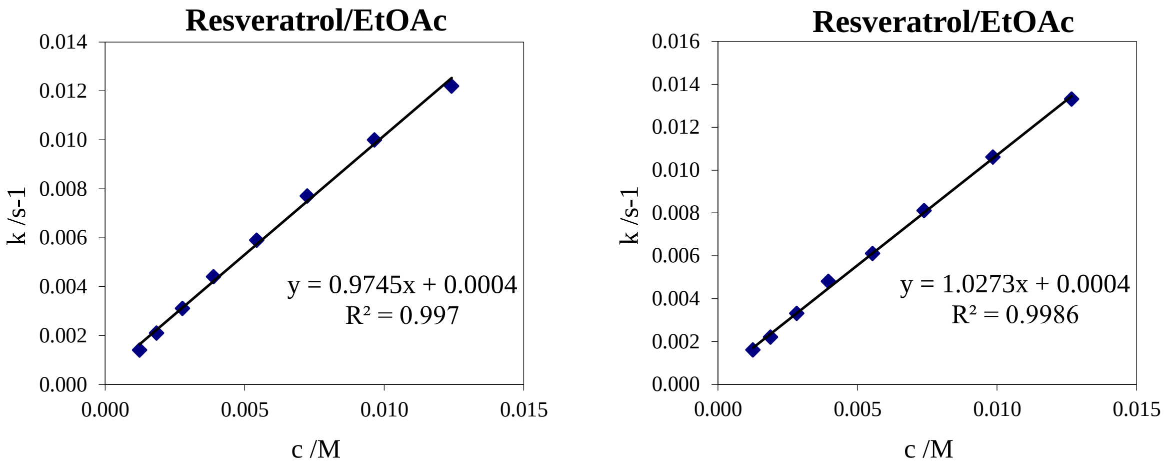

Figure S6. Plots of k_{exp} versus concentration of RSV (data taken from Table S6).

Table S7 Pseudo-first-order rate constant (k_{exp}) for reaction of dpph$^{\bullet}$ (constant initial concentration) with excess of TEMPO$^{\bullet}$ + RSV, 1:1 mol/mol in MeOH and bimolecular rate constant k^{S} calculated as a slope from linear dependence of k_{exp} plotted against concentration of phenol: k_{exp}=k^{S}[RSV] + const, with regression coefficient (R^2) and error $\Delta k^{S}_{90\%}$ calculated as standard deviation for confidential level 90%.

**Neat MeOH**			**Neat MeOH**		
[TEMPO$^{\bullet}$] / mM	[RSV] / mM	k_{exp} / s^{-1}	[TEMPO$^{\bullet}$] / mM	[RSV] / mM	k_{exp} / s^{-1}
10.8	10.8	17.7	10.8	7.01	9.4
8.13	8.13	13.5	8.13	5.26	7.7
6.10	6.10	10.7	6.10	3.94	6.4
4.36	4.36	8.3	4.36	2.82	5.1
3.11	3.11	6.1	3.11	2.01	4.1
2.07	2.07	4.4	2.07	1.34	2.9
1.38	1.38	3.0	1.38	0.89	2.4
0.83	0.83	2.1	0.83	0.54	1.8
k^{s} = 1543 $M^{-1}s^{-1}$<br>$\Delta k^{S}_{90\%}$ = 36<br>R^2 = 0.9977			k^{s} = 1179 $M^{-1}s^{-1}$<br>$\Delta k^{S}_{90\%}$ = 56<br>R^2 = 0.9905		

k^{MeOH} = 1360 ± 260 $M^{-1}s^{-1}$

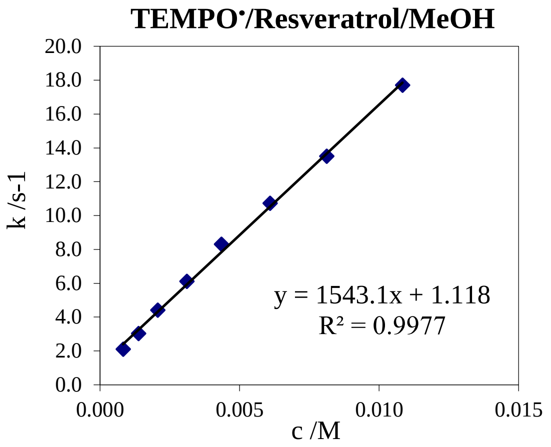

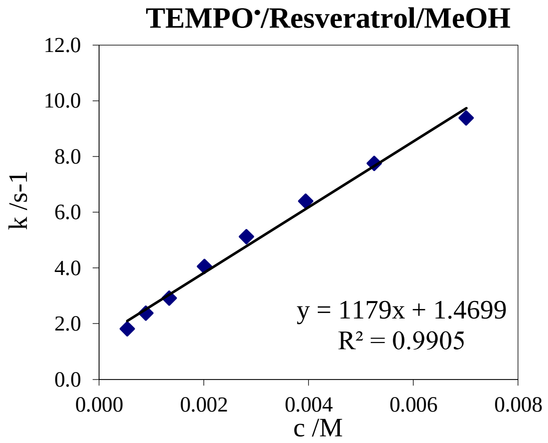

Figure S7. Plots of k_{exp} versus concentration of RSV (data taken from Table S7).

Table S8 Pseudo-first-order rate constant (k_{exp}) for reaction of dpph$^{\bullet}$ (constant initial concentration) with excess of TEMPO$^{\bullet}$ + RSV, 1:1 mol/mol in EtOAc and bimolecular rate constant k^S calculated as a slope from linear dependence of k_{exp} plotted against concentration of phenol: $k_{exp}=k^S$[RSV] + const, with regression coefficient (R^2) and error $\Delta k^S_{90\%}$ calculated as standard deviation for confidential level 90%.

Neat EtOAc			Neat EtOAc		
[TEMPO$^{\bullet}$] / mM	[RSV] / mM	$k_{exp} \times 10^3$ / s^{-1}	[TEMPO$^{\bullet}$] / mM	[RSV] / mM	$k_{exp} \times 10^3$ / s^{-1}
10.5	10.5	9.5	10.5	1.13	12.7
8.14	8.14	7.7	8.14	8.76	9.8
6.11	6.11	6.1	6.11	6.57	7.1
4.58	4.58	4.5	4.58	4.93	5.3
3.27	3.27	3.8	3.27	3.52	4.7
2.34	2.34	2.6	2.34	2.51	2.9
1.56	1.56	1.4	1.56	1.68	2.6
1.04	1.04	1.4	1.04		
k^s = 0.88 $M^{-1}s^{-1}$<br>$\Delta k^S_{90\%}$ = 0.06<br>R^2 = 0.9918			k^s = 1.06 $M^{-1}s^{-1}$<br>$\Delta k^S_{90\%}$ = 0.09<br>R^2 = 0.9906		

$\boldsymbol{k^{EtOAc} = 0.97 \pm 0.13\ M^{-1}s^{-1}}$

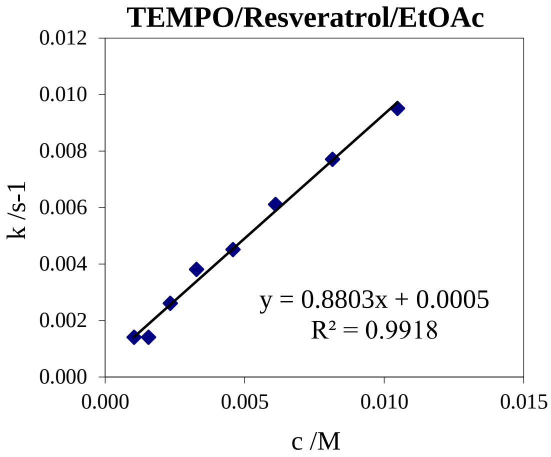

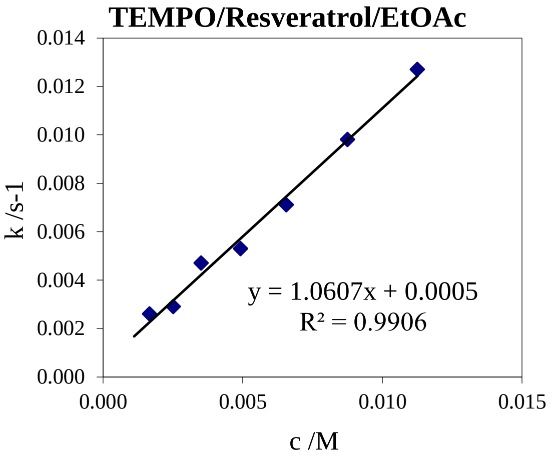

Figure S8. Plots of k_{exp} versus concentration of RSV (data taken from Table S8).

Table S9 Pseudo-first-order rate constant (k_{exp}) for reaction of dpph$^•$ (constant initial concentration) with excess of 4-OH-TEMPO$^•$ + RSV, 1:1 mol/mol in MeOH and bimolecular rate constant k^S calculated as a slope from linear dependence of k_{exp} plotted against concentration of phenol: $k_{exp}=k^S$[RSV] + const, with regression coefficient (R^2) and error $\Delta k^S_{90\%}$ calculated as standard deviation for confidential level 90%.

**Neat MeOH**			**Neat MeOH**		
[4-OH-TEMPO$^•$] / mM	[RSV] / mM	k_{exp} / s^{-1}	[4-OH-TEMPO$^•$] / mM	[RSV] / mM	k_{exp} / s^{-1}
9.13	9.13	5.3	9.73	9.73	5.4
7.10	7.10	4.1	7.57	7.57	4.1
5.33	5.33	2.9	5.68	5.68	3.0
4.00	4.00	2.1	4.26	4.26	2.0
2.85	2.85	1.3	3.04	3.04	1.3
2.04	2.04	1.0	2.17	2.17	1.0
1.36	1.36	0.7	1.45	1.45	0.7
0.91	0.91	0.5	0.97	0.97	0.5
k^s = 591 $M^{-1}s^{-1}$			k^s = 568 $M^{-1}s^{-1}$		
$\Delta k^S_{90\%}$ = 32			$\Delta k^S_{90\%}$ = 28		
R^2 = 0.9951			R^2 = 0.9959		

$\mathbf{k^{MeOH} = 580 \pm 16\ M^{-1}s^{-1}}$

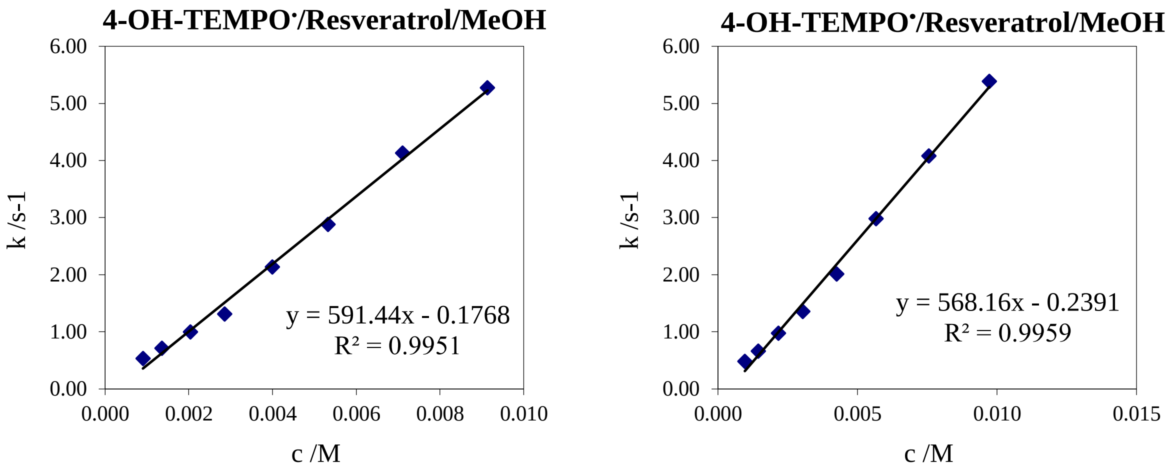

Figure S9. Plots of k_{exp} versus concentration of RSV (data taken from Table S9).

Table S10 Pseudo-first-order rate constant (k_{exp}) for reaction of dpph$^•$ (constant initial concentration) with excess of 4-OH-TEMPO$^•$ + RSV, 1:1 mol/mol in EtOAc and bimolecular rate constant k^S calculated as a slope from linear dependence of k_{exp} plotted against concentration of phenol: $k_{exp}=k^S$[RSV] + const, with regression coefficient (R^2) and error $\Delta k^S_{90\%}$ calculated as standard deviation for confidential level 90%.

Neat EtOAc			Neat EtOAc		
[4-OH-TEMPO$^•$] / mM	[RSV] / mM	$k_{exp} \times 10^3$ / s^{-1}	[4-OH-TEMPO$^•$] / mM	[RSV] / mM	$k_{exp} \times 10^3$ / s^{-1}
10.0	10.0	12.0	9.46	9.46	10.8
7.78	7.78	8.6	7.36	7.36	8.7
5.84	5.84	6.5	5.52	5.52	6.4
4.38	4.38	4.4	4.14	4.14	4.3
3.13	3.13	3.5	2.96	2.96	2.6
2.23	2.23	2.0	1.41	1.41	1.4
1.49	1.49	1.1	0.94	0.94	1.2
0.99	0.99	0.8			
$k^s = 1.23\ M^{-1}s^{-1}$			$k^s = 1.18\ M^{-1}s^{-1}$		
$\Delta k^S_{90\%} = 0.06$			$\Delta k^S_{90\%} = 0.09$		
$R^2 = 0.9954$			$R^2 = 0.9913$		

$$\boldsymbol{K^{EtOAc} = 1.21 \pm 0.04\ M^{-1}s^{-1}}$$

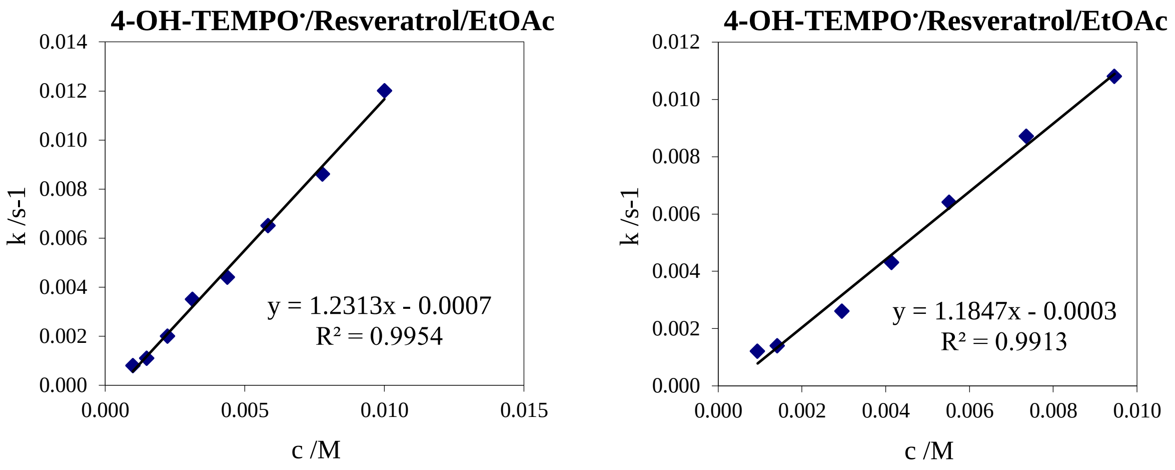

Figure S10. Plots of k_{exp} versus concentration of RSV (data taken from Table S10).

Table S11. Pseudo-first-order rate constant (k_{exp}) for reaction of dpph$^\bullet$ (constant initial concentration) with excess of 3,5-DHA in MeOH and bimolecular rate constant k^S calculated as a slope from linear dependence of k_{exp} plotted against concentration of phenol: $k_{exp}=k^S$[3,5-DHA] + const, with regression coefficient (R^2) and error $\Delta k^S_{90\%}$ calculated as standard deviation for confidential level 90%.

**Neat MeOH**		**Neat MeOH**	
[3,5-DHA] / mM	$k_{exp} \times 10^3$ / s^{-1}	[3,5-DHA] / mM	$k_{exp} \times 10^3$ / s^{-1}
10.2	10.2	13.1	11.2
7.62	8.4	9.82	9.4
5.71	7.1	7.37	8.2
2.91	5.2	5.52	7.3
1.94	4.8	3.95	6.3
1.30	4.2	1.88	4.4
k^s = 0.67 $M^{-1}s^{-1}$<br>$\Delta k^S_{90\%}$ = 0.03<br>R^2 = 0.9984		k^s = 0.58 $M^{-1}s^{-1}$<br>$\Delta k^S_{90\%}$ = 0.07<br>R^2 = 0.9781	
$\mathbf{k^{MeOH} = 0.63 \pm 0.06\ M^{-1}s^{-1}}$			

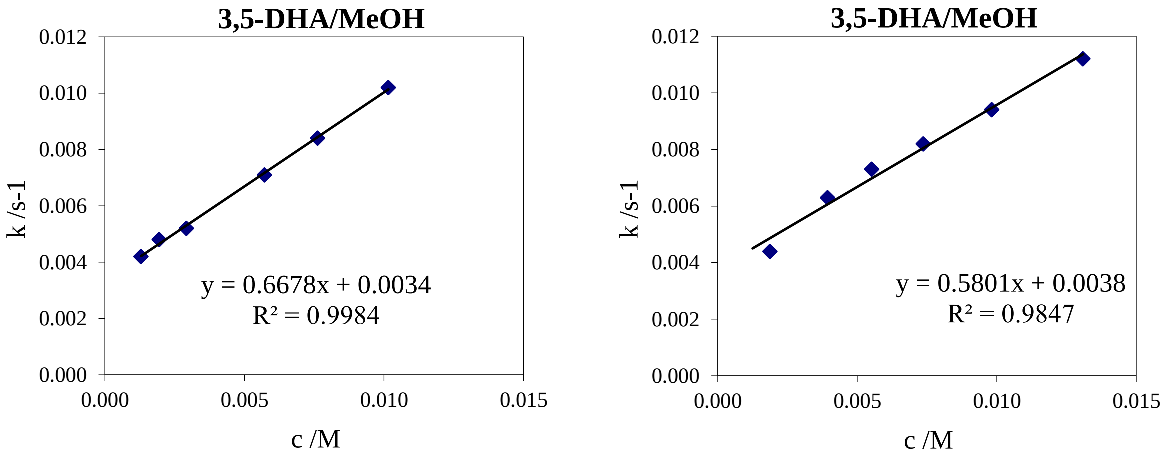

Figure S11. Plots of k_{exp} versus concentration of 3,5-DHA (data taken from Table S11).

Table S12 Pseudo-first-order rate constant (k_{exp}) for reaction of dpph$^{\bullet}$ (constant initial concentration) with excess of TEMPO$^{\bullet}$ + 3,5-DHA, 1:1 mol/mol in MeOH and bimolecular rate constant k^{S} calculated as a slope from linear dependence of k_{exp} plotted against concentration of phenol: $k_{exp}=k^{S}$[3,5-DHA] + const, with regression coefficient (R^2) and error $\Delta k^{S}_{90\%}$ calculated as standard deviation for confidential level 90%.

**Neat MeOH**			**Neat MeOH**		
[TEMPO$^{\bullet}$] / mM	[3,5-DHA] / mM	k_{exp} / s^{-1}	[TEMPO$^{\bullet}$] / mM	[3,5-DHA] / mM	k_{exp} / s^{-1}
12.0	12.0	1.50	11.9	11.9	1.42
9.4	9.4	1.11	9.3	9.3	1.12
5.3	5.3	0.73	4.7	4.7	0.75
3.6	3.6	0.55	3.2	3.2	0.54
2.6	2.6	0.46	2.3	2.3	0.42
1.7	1.7	0.38	1.5	1.5	0.31
1.2	1.2	0.23	1.0	1.0	0.22
k^{s} = 108.4 $M^{-1}s^{-1}$<br>$\Delta k^{S}_{90\%}$ = 8.1<br>R^2 = 0.9923			k^{s} = 106.3 $M^{-1}s^{-1}$<br>$\Delta k^{S}_{90\%}$ = 9.1<br>R^2 = 0.9902		

$\boldsymbol{k^{MeOH} = 107.4 \pm 1.5\ M^{-1}s^{-1}}$

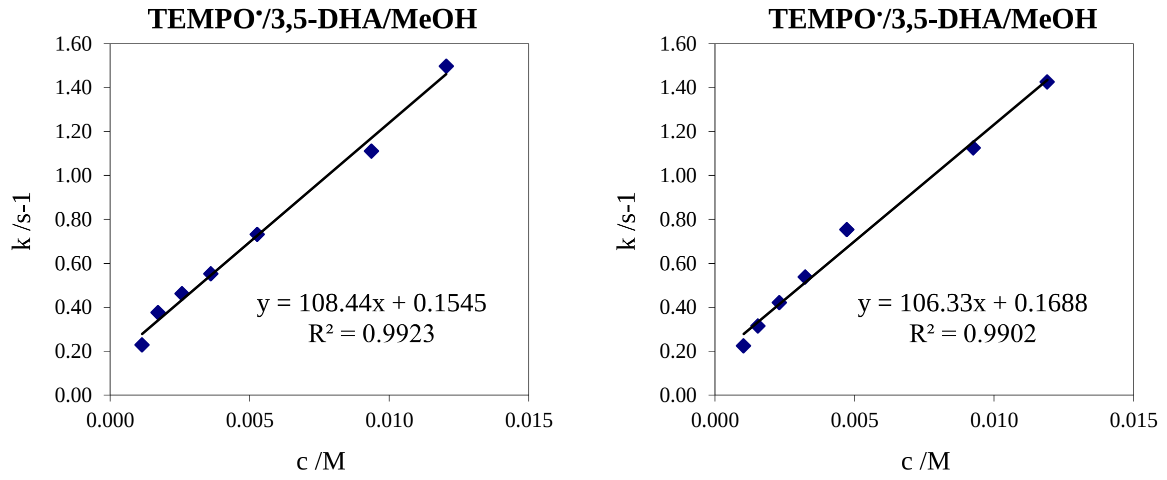

Figure S12. Plots of k_{exp} versus concentration of 3,5-DHA (data taken from Table S12).

Table S13. Pseudo-first-order rate constant (k_{exp}) for reaction of dpph$^•$ (constant initial concentration) with excess of TEMPO$^•$ in acidified MeOH and bimolecular rate constant k^S calculated as a slope from linear dependence of k_{exp} plotted against concentration of nitroxide: $k_{exp}=k^S$[TEMPO$^•$] + const, with regression coefficient (R^2) and error $\Delta k^S_{90\%}$ calculated as standard deviation for confidential level 90%.

**MeOH / 10 mM AcOH**		**MeOH / 10 mM AcOH**	
[TEMPO$^•$] / mM	k_{exp} / s^{-1}	[TEMPO$^•$] / mM	k_{exp} / s^{-1}
12.5	1.7	12.3	1.8
9.36	1.4	9.22	1.4
7.02	1.1	6.91	1.1
5.01	0.9	4.94	0.9
3.58	0.7	3.53	0.7
2.39	0.4	2.35	0.5
1.59	0.4	1.57	0.3
0.96	0.3		
k^s = 130.2 $M^{-1}s^{-1}$		k^s = 133.5 $M^{-1}s^{-1}$	
$\Delta k^S_{90\%}$ = 6.7		$\Delta k^S_{90\%}$ = 5.9	
R^2 = 0.9955		R^2 = 0.9781	
$\boldsymbol{k^{MeOH}}$ **= 132 ± 2** $\boldsymbol{M^{-1}s^{-1}}$			

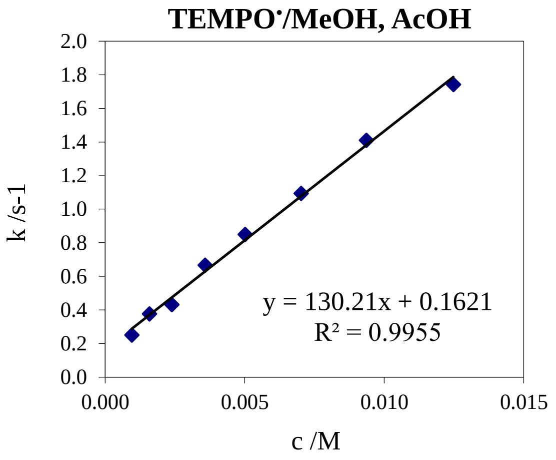

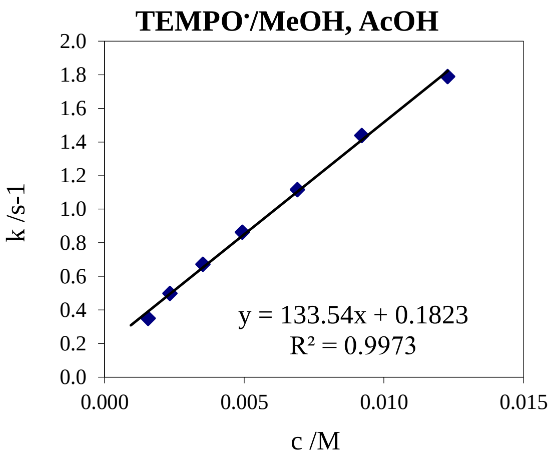

Figure S13. Plots of k_{exp} versus concentration of TEMPO$^•$ (data taken from Table S13).

Table S14. Pseudo-first-order rate constant (k_{exp}) for reaction of dpph$^•$ (constant initial concentration) with excess of 4-OH-TEMPO$^•$ in acidified MeOH and bimolecular rate constant k^S calculated as a slope from linear dependence of k_{exp} plotted against concentration of nitroxide: k_{exp}=k^S[4-OH-TEMPO$^•$] + const, with regression coefficient (R^2) and error $\Delta k^S_{90\%}$ calculated as standard deviation for confidential level 90%.

**MeOH / 10 mM AcOH**		**MeOH / 10 mM AcOH**	
[4-OH-TEMPO$^•$] / mM	k_{exp} / s^{-1}	[4-OH-TEMPO$^•$] / mM	k_{exp} / s^{-1}
8.90	0.19	12.9	0.23
6.67	0.15	10.1	0.20
5.00	0.13	7.55	0.16
3.57	0.10	5.66	0.14
2.55	0.08	4.05	0.12
1.70	0.06	2.89	0.11
1.22	0.06	1.93	0.09
k^s = 18.3 $M^{-1}s^{-1}$		k^s = 12.9 $M^{-1}s^{-1}$	
$\Delta k^S_{90\%}$ = 1.1		$\Delta k^S_{90\%}$ = 1.0	
R^2 = 0.9954		R^2 = 0.9914	

k^{MeOH} **= 15.6 ± 3.8** $M^{-1}s^{-1}$

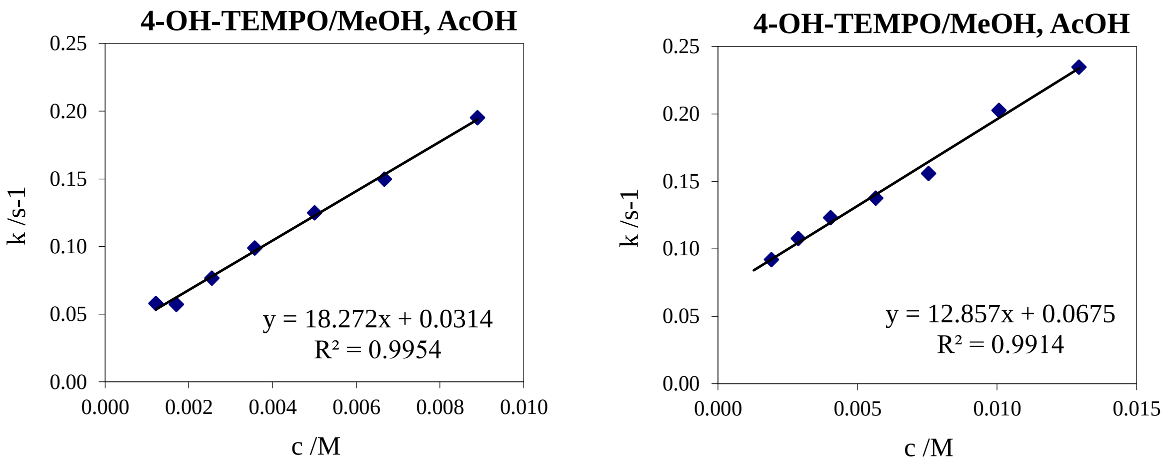

Figure S14. Plots of k_{exp} versus concentration of 4-OH-TEMPO$^•$ (data taken from Table S14).

Table S15. Pseudo-first-order rate constant (k_{exp}) for reaction of dpph$^{\bullet}$ (constant initial concentration) with excess of RSV in acidified MeOH and bimolecular rate constant k^S calculated as a slope from linear dependence of k_{exp} plotted against concentration of phenol: $k_{exp}=k^S$[RSV] + const, with regression coefficient (R^2) and error $\Delta k^S_{90\%}$ calculated as standard deviation for confidential level 90%.

**MeOH / 10 mM AcOH**		**MeOH / 10 mM AcOH**	
[RSV] / mM	$k_{exp} \times 10^3$ / s^{-1}	[RSV] / mM	$k_{exp} \times 10^3$ / s^{-1}
12.2	38.9	12.2	33.0
9.47	31.2	9.52	30.3
7.10	25.8	7.14	24.0
5.33	20.7	3.83	16.7
3.81	17.5	2.73	13.9
2.72	12.8	1.21	10.9
1.81	12.2		
1.21	9.9		
k^s = 2.6 $M^{-1}s^{-1}$		k^s = 2.1 $M^{-1}s^{-1}$	
$\Delta k^S_{90\%}$ = 0.1		$\Delta k^S_{90\%}$ = 0.2	
R^2 = 0.9965		R^2 = 0.9877	
**k^{MeOH} = 2.4 ± 0.4 $M^{-1}s^{-1}$**			

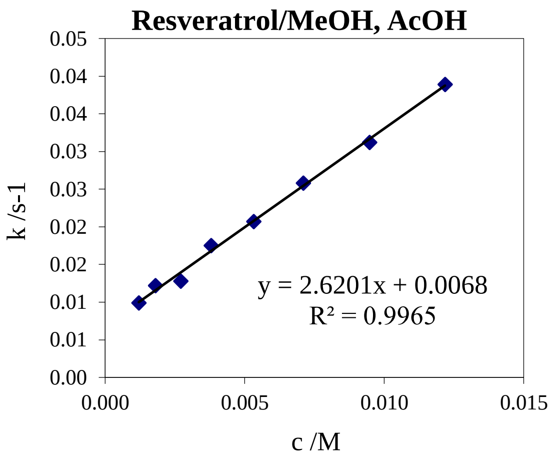

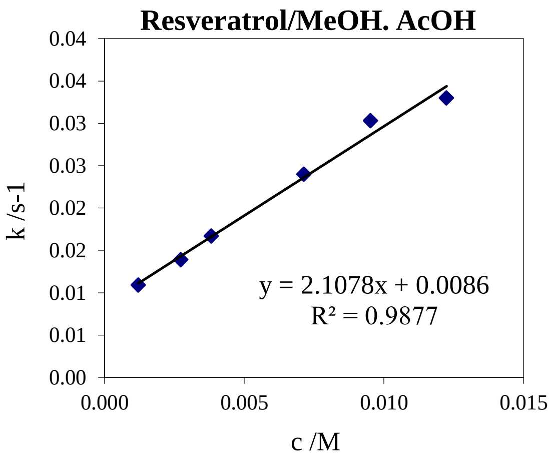

Figure S15. Plots of k_{exp} versus concentration of RSV (data taken from Table S15).

Table S16. Pseudo-first-order rate constant (k_{exp}) for reaction of dpph$^•$ (constant initial concentration) with excess of TEMPO$^•$ in MeOH with 10 mM HFIP and bimolecular rate constant k^S calculated as a slope from linear dependence of k_{exp} plotted against concentration of nitroxide: $k_{exp}=k^S[TEMPO^•]$ + const, with regression coefficient (R^2) and error $\Delta k^S_{90\%}$ calculated as standard deviation for confidential level 90%.

**MeOH / 10 mM HFIP**		**MeOH / 10 mM HFIP**	
[TEMPO$^•$] / mM	k_{exp} / s^{-1}	[TEMPO$^•$] / mM	k_{exp} / s^{-1}
11.9	1.07	12.0	1.15
9.3	0.81	9.3	0.89
6.9	0.78	5.2	0.69
5.2	0.60	3.7	0.66
3.7	0.54	2.7	0.56
2.7	0.48	1.8	0.50
1.2	0.35		
k^s = 63.3 $M^{-1}s^{-1}$<br>$\Delta k^S_{90\%}$ = 8.7<br>R^2 = 0.9745		k^s = 59.3 $M^{-1}s^{-1}$<br>$\Delta k^S_{90\%}$ = 9.2<br>R^2 = 0.9751	

$\boldsymbol{k^{MeOH}}$ **= 61.3 ± 2.8** $\boldsymbol{M^{-1}s^{-1}}$

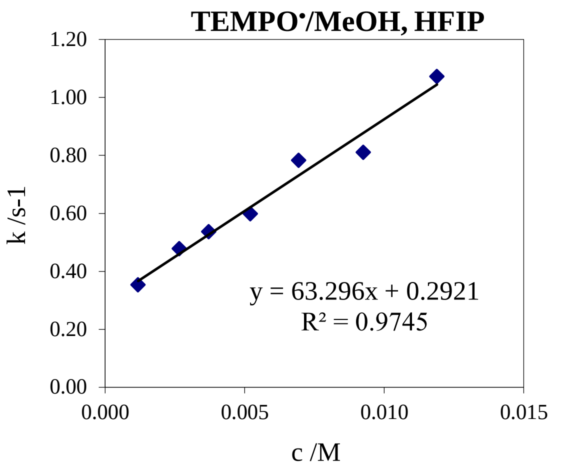

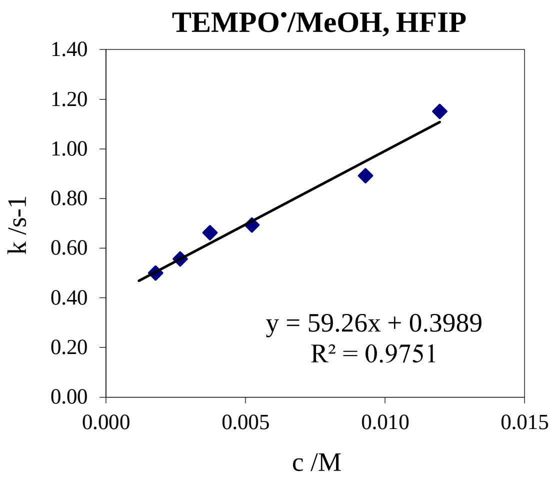

Figure S16. Plots of k_{exp} versus concentration of TEMPO$^•$ (data taken from Table S16).

Table S17. Pseudo-first-order rate constant (k_{exp}) for reaction of dpph$^{\bullet}$ (constant initial concentration) with excess of TEMPO-H in MeOH and bimolecular rate constant k^S calculated as a slope from linear dependence of k_{exp} plotted against concentration of TEMPO-H: $k_{exp}=k^S$[TEMPO-H] + const, with regression coefficient (R^2) and error $\Delta k^S_{90\%}$ calculated as standard deviation for confidential level 90%.

**Neat MeOH**		**Neat MeOH**	
[TEMPO-H] / mM	k_{exp} / s^{-1}	[TEMPO-H] / mM	k_{exp} / s^{-1}
5.2	0.24	5.2	0.20
4.1	0.19	4.0	0.17
3.1	0.15	3.1	0.14
2.3	0.13	2.3	0.11
1.6	0.11	1.5	0.09
1.2	0.09	1.2	0.08
0.8	0.08	0.8	0.05
		0.5	0.04
k^s = 35.0 M^{-1}s^{-1}		k^s = 34.0M^{-1}s^{-1}	
$\Delta k^S_{90\%}$ = 2.0		$\Delta k^S_{90\%}$ = 3.0	
R^2 = 0.9966		R^2 = 0.9751	

k^{MeOH} = 34.5 ± 0.5 M^{-1}s^{-1}

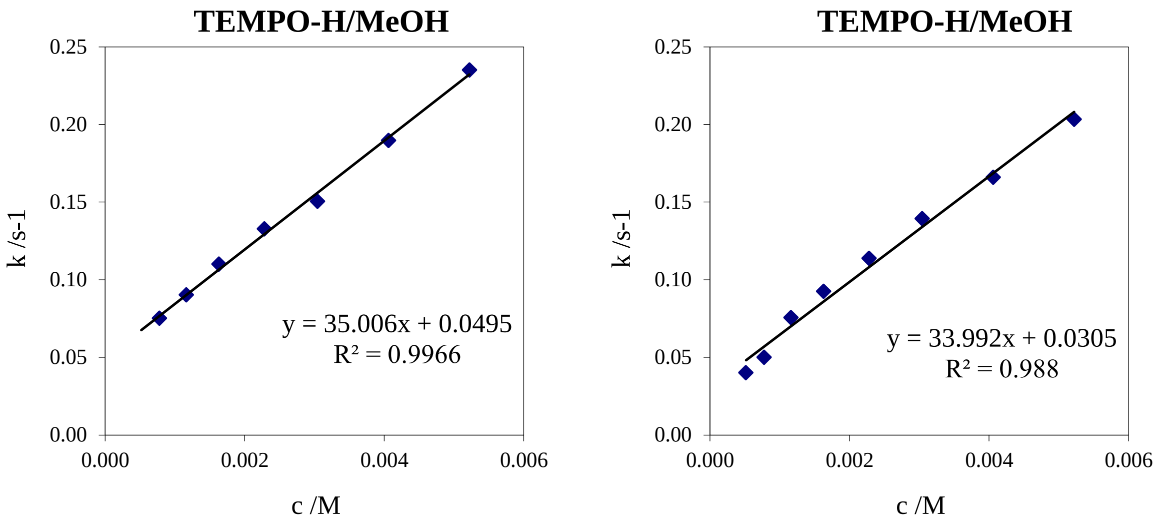

Figure S17. Plots of k_{exp} versus concentration of TEMPO-H (data taken from Table S17).

Table S18. Pseudo-first-order rate constant (k_{exp}) for reaction of dpph$^{\bullet}$ (constant initial concentration) with excess of TEMPO-H in acidified MeOH and bimolecular rate constant k^S calculated as a slope from linear dependence of k_{exp} plotted against concentration of TEMPO-H: $k_{exp}=k^S$[TEMPO-H] + const, with regression coefficient (R^2) and error $\Delta k^S_{90\%}$ calculated as standard deviation for confidential level 90%.

**MeOH / 10 mM AcOH**		**MeOH / 10 mM AcOH**	
[TEMPO-H] / mM	k_{exp} / s^{-1}	[TEMPO-H] / mM	k_{exp} / s^{-1}
5.4	0.28	6.5	0.29
4.2	0.23	3.8	0.19
2.4	0.14	2.9	0.15
1.7	0.12	2.0	0.12
1.2	0.09	1.5	0.09
0.8	0.07	1.9	0.07
0.5	0.05	0.6	0.04
k^s = 46 $M^{-1}s^{-1}$<br>$\Delta k^S_{90\%}$ = 3.0<br>R^2 = 0.9958		k^s = 41 $M^{-1}s^{-1}$<br>$\Delta k^S_{90\%}$ = 3.0<br>R^2 = 0.9908	
**k^{MeOH} = 43.5 ± 3.5 $M^{-1}s^{-1}$**			

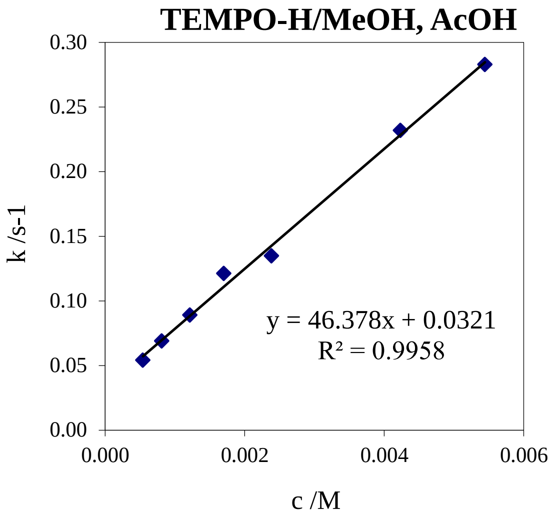

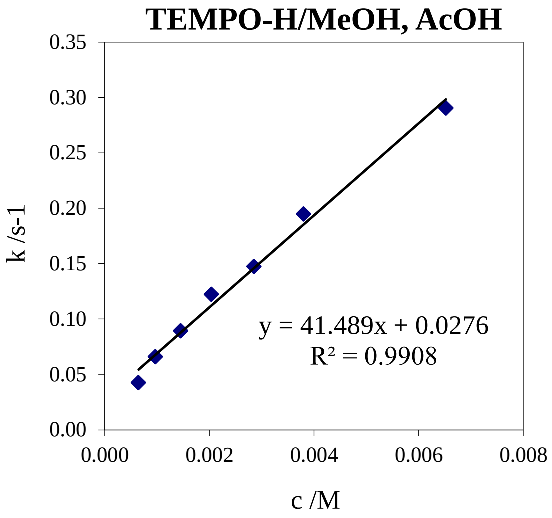

Figure S18. Plots of k_{exp} versus concentration of TEMPO-H (data taken from Table S18).

Table S19. Pseudo-first-order rate constant (k_{exp}) for reaction of dpph$^\bullet$ (constant initial concentration) with excess of TEMPO-H in EtOAc and bimolecular rate constant k^S calculated as a slope from linear dependence of k_{exp} plotted against concentration of TEMPO-H: $k_{exp}=k^S$[TEMPO-H] + const, with regression coefficient (R^2) and error $\Delta k^S_{90\%}$ calculated as standard deviation for confidential level 90%.

**Neat EtOAc**		**Neat EtOAc**	
[TEMPO-H] / mM	$k_{exp} \times 10^2$ / s^{-1}	[TEMPO-H] / mM	$k_{exp} \times 10^2$ / s^{-1}
4.9	4.63	5.2	3.22
3.8	3.51	4.0	2.60
2.9	2.71	3.0	1.89
2.1	2.05	2.3	1.51
1.5	1.52	1.6	1.10
1.1	1.14	1.2	0.82
0.7	0.67	0.7	0.51
0.5	0.48	0.5	0.38
k^s = 9.3 $M^{-1}s^{-1}$		k^s = 6.1 $M^{-1}s^{-1}$	
$\Delta k^S_{90\%}$ = 0.3		$\Delta k^S_{90\%}$ = 0.2	
R^2 = 0.9987		R^2 = 0.9985	

$\boldsymbol{k^{MeOH} = 7.7 \pm 2.2\ M^{-1}s^{-1}}$

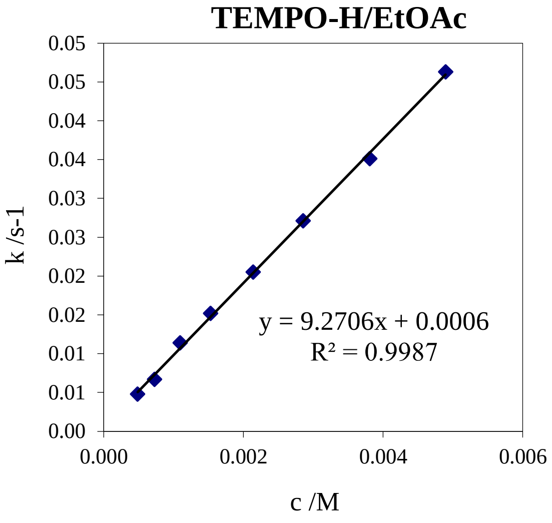

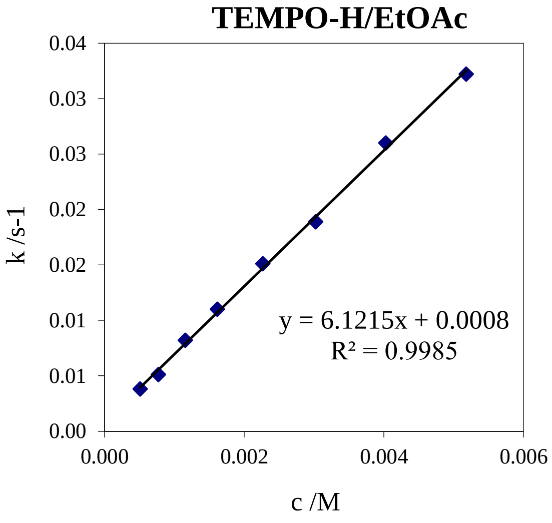

Figure S19. Plots of k_{exp} versus concentration of TEMPO-H (data taken from Table S19).

Table S20. Pseudo-first-order rate constant (k_{exp}) for reaction of dpph$^•$ (constant initial concentration) with excess of RSV + TEMPO-H, 1:1 mol/mol in MeOH and bimolecular rate constant k^S calculated as a slope from linear dependence of k_{exp} plotted against concentration of phenol: $k_{exp}=k^S$[RSV] + const, with regression coefficient (R^2) and error $\Delta k^S_{90\%}$ calculated as standard deviation for confidential level 90%.

**Neat MeOH**			**Neat MeOH**		
[TEMPO-H] / mM	[RSV] / mM	k_{exp} / s^{-1}	[TEMPO-H] / mM	[RSV] / mM	k_{exp} / s^{-1}
10.4	10.4	20.2	10.1	10.1	21.3
8.1	8.1	16.0	7.9	7.9	16.8
6.1	6.1	11.7	5.9	5.9	12.4
4.6	4.6	8.4	4.4	4.4	9.0
3.3	3.3	5.8	3.2	3.2	6.0
2.3	2.3	3.8	2.3	2.3	4.2
1.6	1.6	2.2	1.5	1.5	2.5
1.0	1.0	1.0	1.0	1.0	1.5
k^s = 2048 $M^{-1}s^{-1}$<br>$\Delta k^S_{90\%}$ = 37<br>R^2 = 0.9994			k^s = 2196 $M^{-1}s^{-1}$<br>$\Delta k^S_{90\%}$ = 37<br>R^2 = 0.9995		
$\boldsymbol{K^{MeOH}}$ **= 2122 ± 105 $\boldsymbol{M^{-1}s^{-1}}$**					

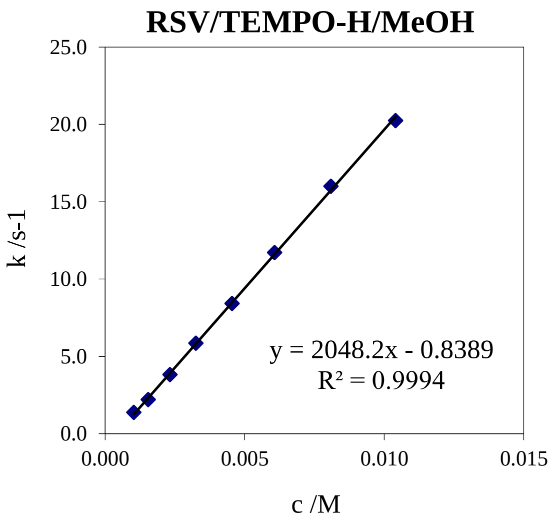

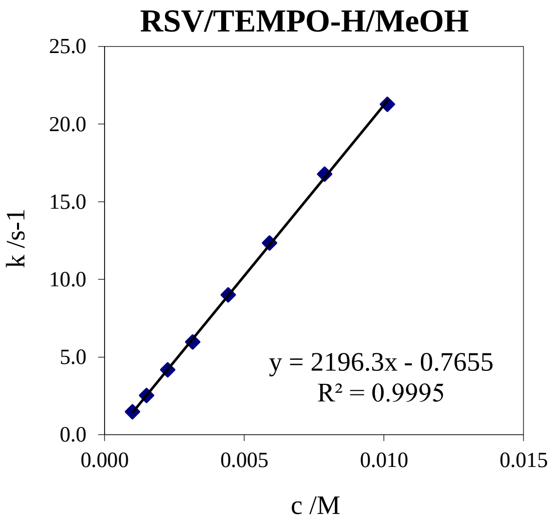

Figure S20. Plots of k_{exp} versus concentration of RSV (data taken from Table S20).

Table S21. Pseudo-first-order rate constant (k_{exp}) for reaction of dpph$^{\bullet}$ (constant initial concentration) with excess of RSV + TEMPO-H, 1:1 mol/mol in EtOAc and bimolecular rate constant k^S calculated as a slope from linear dependence of k_{exp} plotted against concentration of phenol: $k_{exp}=k^S$[RSV] + const, with regression coefficient (R^2) and error $\Delta k^S_{90\%}$ calculated as standard deviation for confidential level 90%.

**Neat EtOAc**			**Neat EtOAc**		
[TEMPO-H] / mM	[RSV] / mM	$k_{exp} \times 10^2$ / s^{-1}	[TEMPO-H] / mM	[RSV] / mM	$k_{exp} \times 10^2$ / s^{-1}
9.8	9.8	7.0	9.6	9.6	10.2
7.6	7.6	5.4	7.5	7.5	7.7
5.7	5.7	3.8	5.6	5.6	5.7
4.3	4.3	2.8	4.2	4.2	4.2
3.1	3.1	2.0	3.0	3.0	2.9
2.2	2.2	1.4	2.2	2.2	2.1
1.5	1.5	0.9	1.4	1.4	1.3
1.0	1.0	0.7	0.9	0.9	0.9
k^s = 7.3 $M^{-1}s^{-1}$			k^s = 10.7 $M^{-1}s^{-1}$		
$\Delta k^S_{90\%}$ = 0.2			$\Delta k^S_{90\%}$ = 0.2		
R^2 = 0.9981			R^2 = 0.9994		

$\boldsymbol{K^{EtOAc} = 9.0 \pm 2.4\ M^{-1}s^{-1}}$

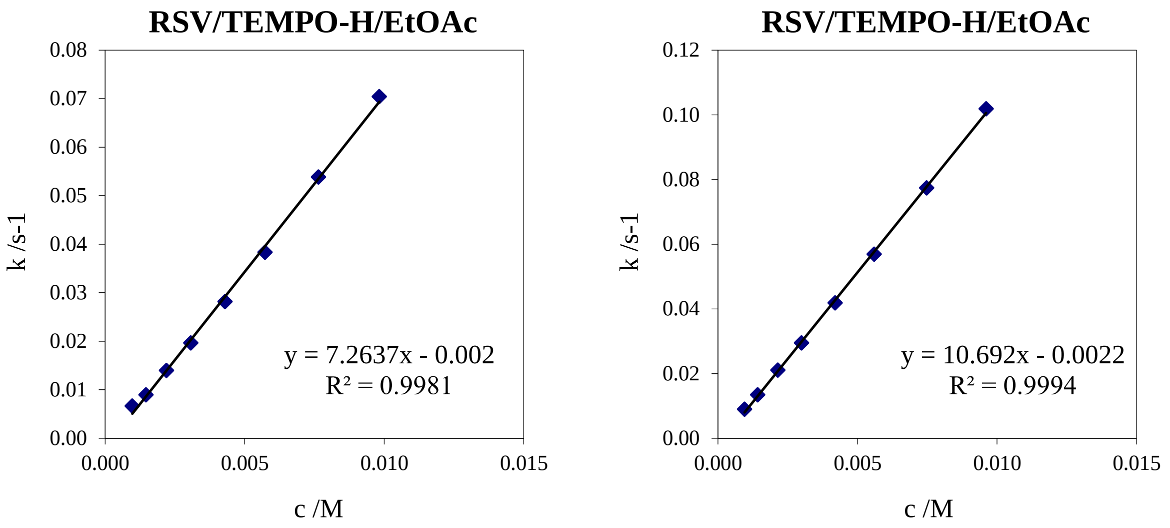

Figure S21. Plots of k_{exp} versus concentration of RSV (data taken from Table S21).

Table S22. Literature values of oxidation and reduction potentials (vs NHE) for TEMPO•, 4-OH-TEMPO•, TEMPOH•+, TEMPOnium+, resveratrol and dpph•.

	E_{ox} /mV	E_{red} / mV
TEMPO•	750±5 [1], 734 [a], 864 [b], 730-750 (pH7)[c] 804 (pH9.3) [d]	207,[e] 213[f]
4-OH-TEMPO•	818[2,]806[3], 830*[4]	229,[e] 233[f]
TEMPOH•+		955 ± 15 [g]
TEMPOnium+		750± 5,[1] 804 (pH 9.3)[h]
RSV	849 (pH3.2), ~650 (pH7.0)[i]	
dpph•		545[j], 424[k]

[a] In water, pH 7.[5]

[b] Cyclic voltametry 0.003 M TEMPO, in MeOH/MeCN (1:1), 0.1 M Bu_4NBF_4, half-wave potential (620mV vs. SCE),[6] here recalculated to NHE.

[c] Values collected by Tikhonov et al. [2, 7]

[d] +560V vs. SCE in *t*-butanol/water (1:1). [8]

[e] Reversible half-wave potential of the reduction of TEMPO (or 4-OH-TEMPO) at pH 7 determined experimentally by Kato et al.[9]

[f] The resulting half-wave potentials (versus SHE) for one electron oxidation and reduction potentials calculated theoretically by Hodgson et al. [10] of several nitroxides.

[g] Standard reduction potential for TEMPOH•+ /TEMPOH pair calculated from $E^o_{(TEMPOnium/TEMPO\bullet)}$ and equilibrium constant for TEMPOH•+ + TEMPO• ⇆ TEMPOnium +TEMPOH. [1]

[h] -0.65V 0.56 vs. SCE in t-butanol/water (1:1). [8]

[i] In ethanol/water, measured versus Ag/AgCl and recalculated into NHE. Those potentials correspond to the oxidation of the phenol moiety (the slope is - 45 mV/pH), the oxidation of the resorcinol group occurs at potential ca 200mV higher and the slope is -59mV/pH.[11]

[j] For dpph•/dpph⁻,H+ redox pair in water-methanol 1:1 at pH =7.[12]

[k] In acetonitrile.[13]

REFERENCES

1. V. D. Sen and V. A. Golubev, *J Phys Org Chem*, 2009, **22**, 138-143.
2. V. D. Sen', I. V. Tikhonov, L. I. Borodin, E. M. Pliss, V. A. Golubev, M. A. Syroeshkin and A. I. Rusakov, *J. Phys. Org. Chem.*, 2015, **28**, 17-24.
3. T. Yamasaki, Y. Matsuoka, F. Mito, M. Yamato and K. i. Yamada, *Asian J. Org. Chem.*, 2013, **2**, 388-391.
4. A. Orita, M. G. Verde, M. Sakai and Y. S. Meng, *J. Power Source*, 2016, **321**, 126-134.
5. J. R. Fish, S. G. Swarts, M. D. Sevilla and T. Malinski, *J. Phys. Chem.*, 1988, **92**, 3745-3751.
6. X.-Y. Qian, S.-Q. Li, J. Song and H.-C. Xu, *ACS Catalysis*, 2017, **7**, 2730-2734.
7. I. V. Tikhonov, V. D. Sen', L. I. Borodin, E. M. Pliss, V. A. Golubev and A. I. Rusakov, *J. Phys. Org. Chem.*, 2014, **27**, 114-120.
8. R. A. Green, J. T. Hill-Cousins, R. C. D. Brown, D. Pletcher and S. G. Leach, *Electrochim. Acta*, 2013, **113**, 550-556.
9. Y. Kato, Y. Shimizu, L. Yijing, K. Unoura, H. Utsumi and T. Ogata, *Electrochim. Acta*, 1995, **40**, 2799-2802.
10. J. L. Hodgson, M. Namazian, S. E. Bottle and M. L. Coote, *J. Phys. Chem. A*, 2007, **111**, 13595-13605.
11. O. Corduneanu, P. Janeiro and A. M. O. Brett, *Electroanalysis*, 2006, **18**, 757-762.
12. Q.-k. Zhuang, F. Scholz and F. Pragst, *Electrochem. Comm.*, 1999, **1**, 406-410.
13. I. Nakanishi, K. Fukuhara, T. Shimada, K. Ohkubo, Y. Iizuka, K. Inami, M. Mochizuki, S. Urano, S. Itoh and N. Miyata, *J. Chem. Soc. Perkin 2*, 2002, 1520-1524.
